# Characterization of an eye field-like state during optic vesicle organoid development

**DOI:** 10.1242/dev.201432

**Published:** 2023-08-09

**Authors:** Liusaidh J. Owen, Jacqueline Rainger, Hemant Bengani, Fiona Kilanowski, David R. FitzPatrick, Andrew S. Papanastasiou

**Affiliations:** ^1^ MRC Human Genetics Unit, Institute of Genetics and Cancer, University of Edinburgh, Edinburgh EH4 2XU, UK; ^2^ School of Informatics, University of Edinburgh, Edinburgh EH8 9AB, UK

**Keywords:** Eye field specification, Transcription factors, Gene regulatory networks

## Abstract

Specification of the eye field (EF) within the neural plate marks the earliest detectable stage of eye development. Experimental evidence, primarily from non-mammalian model systems, indicates that the stable formation of this group of cells requires the activation of a set of key transcription factors. This crucial event is challenging to probe in mammals and, quantitatively, little is known regarding the regulation of the transition of cells to this ocular fate. Using optic vesicle organoids to model the onset of the EF, we generate time-course transcriptomic data allowing us to identify dynamic gene expression programmes that characterize this cellular-state transition. Integrating this with chromatin accessibility data suggests a direct role of canonical EF transcription factors in regulating these gene expression changes, and highlights candidate *cis*-regulatory elements through which these transcription factors act. Finally, we begin to test a subset of these candidate enhancer elements, within the organoid system, by perturbing the underlying DNA sequence and measuring transcriptomic changes during EF activation.

## Teaser

**Summary:** Analyses of RNA- and ATAC-sequencing measurements of optic-vesicle organoid development robustly characterize the mammalian eye-field and generate specific hypotheses of *cis*-regulation of eye-field transcription factors.

## INTRODUCTION

Vertebrate eye development begins with the formation of the eye field – a patch of morphologically indistinct neuroectodermal cells within the anterior neural plate defined by co-expression of a set of transcription factors (TFs). The eye field was first identified in *Xenopus laevis* embryos (Zuber et al., 2003; Zuber, 2010) and expresses a specific set of TFs, known as the eye-field transcription factors (EFTFs), with those being: *PAX6*, *RAX*, *SIX3*, *SIX6*, *LHX2*, *OTX2*, *NR2E1* and *TBX3* (gene symbols of human orthologues). Mutations in at least three of the genes encoding orthologues of the *Xenopus* EFTFs can cause severe bilateral eye malformations in humans (*OTX2*, *PAX6* and *RAX*) (Fitzpatrick and van Heyningen, 2005). It seems likely that these genes are involved in eye-field specification in humans. The EFTFs are thought to form part of a gene regulatory network (GRN) essential for initiating eye development and the goal of this work is to characterize this GRN in mammals and gain a deeper understanding of how it is initiated and stably maintained.

The eye field has been difficult to study in mammals as it forms in early post-implantation embryos (∼18 post-ovulatory days in humans, approximately embryonic day 7.5 in mouse). The Sasai lab developed a simple defined organoid culture system (Eiraku et al., 2011; Eiraku and Sasai, 2012; Nakano et al., 2012; Sakakura et al., 2016) that supports efficient differentiation of human and mouse embryonic stem cells (ESCs) into early eye structures; optic vesicles (OVs) evaginate from the main body of the organoid and then develop into optic cup structures with partially differentiated neural retina. This transformative technology established that early eye development is a self-organizing system and provides a window onto the hitherto cryptic processes driving the commitment to eye fate in mammals.

We have performed a functional genomic analysis of the eye field-like cells that appear in the very early stages of optic vesicle organoid differentiation. By performing computational analyses of the transcriptomic changes during OV differentiation, we find that establishment of the eye field in mouse ESC-derived OVs involves strong upregulation of a set of genes that includes the same key transcription factors identified in *X. laevis*. Furthermore, by integrating transcriptomic and chromatin-accessibility data, we show that these TFs likely regulate the stable transition of cells to an ocular fate by binding distal enhancers in genomic domains local to EF genes. By examining how chromatin accessibility, associated with key TFs, changes at individual genomic loci, we generate hypotheses of plausible *cis*-regulatory elements (CREs – enhancers and repressors) required for differentiation from pluripotency to eye-field cell states. Finally, we test a subset of these hypotheses by perturbing the underlying DNA sequences. In all, by exploiting the robust and reproducible model system offered by mESC-derived organoids, our study provides a first quantitative understanding of the regulatory mechanisms underlying mammalian eye-field specification.

## RESULTS

### Mammalian eye-field genes overlap significantly with *Xenopus* EFTFs

**Fig. 1.**
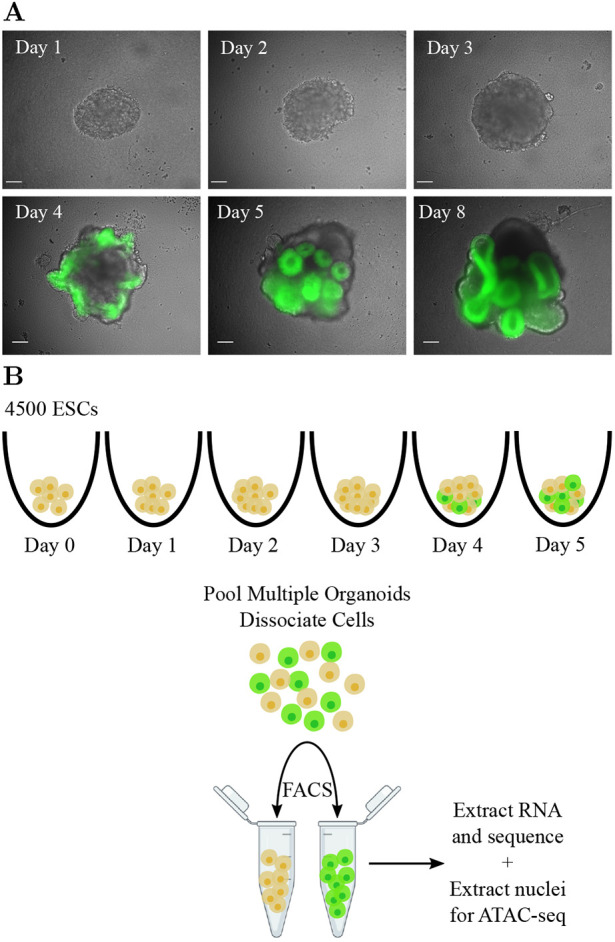
**Exploiting optic vesicle organoids to probe the molecular origins of eye-field specification.** (A) Representative organoids from days 1-5, and day 8. Scale bars: 100 µm. (B) Schematic of the experimental setup for time-course RNA-seq and ATAC-seq data generation.

Mammalian eye-field specification – the crucial first stage of ocular development – is an event that occurs very early in development (during gastrulation), and as such is extremely challenging to study *in vivo*. We have exploited a reproducible, *in vitro* organoid model system enabling us to generate data from this cell-state transition and through computational analysis gain a quantitative understanding of the underlying regulatory mechanisms. We performed OV organoid culture using mouse embryonic stem cells (mESCs) that have a GFP fluorescent reporter cassette knocked into the *Rax* locus (Eiraku and Sasai, 2012). The expression pattern of *Rax* suggests that cells expressing this gene are either competent to become, or are already, retinal progenitors (Furukawa et al., 1997), although *Rax* is also expressed in the hypothalamus (Wataya et al., 2008). Rax-positive cells became detectable between days 3 and 4 in culture (Fig. 1A), and at days 5 and 6 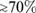 of live cells were Rax positive (Table S1). By day 5, the cell aggregates had developed a 3D structure typical of retinal organoids, as previously described (Sakakura et al., 2016). We performed bulk RNA-sequencing (RNA-seq) analysis using biological triplicates of OV organoid cultures at days 0, 1, 2, 3, 4 and 5. To enrich for cells in an eye-field state, at days 4 and 5 the cells were sorted by fluorescence-activated cell sorting (FACS) into GFP-positive and GFP-negative populations (Fig. 1B).

**Fig. 2.**
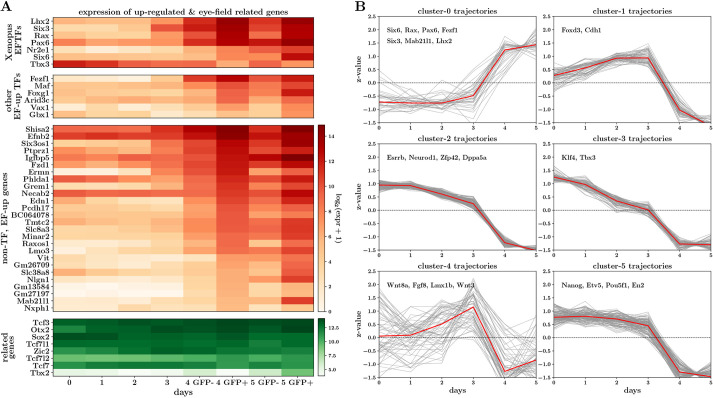
**RNA-seq analysis reveals coordinated upregulation of canonical EFTFs during optic vesicle development.** (A) Heatmap displaying gene expression across the OV time course, for canonical eye-field genes, differentially upregulated (EF-up) genes and genes known to have an important role in early eye development. (B) Clustering of EF-up and EF-down gene trajectories reveals six clusters with three underlying patterns across the time course. At each time point, heatmap and trajectory plots indicate expression averaged across triplicates.

To identify genes characterizing the transition to the eye field, we looked for differentially expressed genes between day 3 and the GFP-positive samples on days 4 and 5 [using DEseq2 (Love et al., 2014); false discovery rate (FDR) <0.001, absolute log2-fold-change >1.5]. This analysis revealed a small set of ‘EF-up’ genes (37) that were significantly upregulated between these time points, of which 11 correspond to known mouse TFs (Fig. 2A). Importantly, these included the canonical EFTF genes *Rax*, *Pax6*, *Lhx2*, *Six3* and *Six6*, indicating that drivers of the optic-fate in mammals include most of the key TFs also discovered in *Xenopus*. *Six6* was expressed slightly later, consistent with evidence that it is a downstream target of *Lhx2* and *Pax6* (Tétreault et al., 2009). Although not sufficiently upregulated to pass our threshold, *Nr2e1* displayed an expression pattern consistent with that of the other canonical EFTFs. The only canonical EFTF with an unexpected expression pattern – one that is anti-correlated with the onset of the eye field – was *Tbx3*. This could be because *Tbx3* plays a different role in mouse than in *Xenopus* (*Tbx3* is a known component of the pluripotency network in mESCs; Russell et al., 2015) or because the *Xenopus Tbx3* orthologue in mouse may not be *Tbx3*. *Tbx2* is the likely candidate, being the only Tbx family member for which expression is highly correlated with onset of the eye field (Fig. 2A). Finally, as expected, when inspecting genes differentially upregulated specifically between the GFP-positive and -negative cells at day 5, we found markers of later eye development, including *Vsx2*, *Vax1*, *Mitf* and *Aldh1a3* (Fig. S2C). This provides transcriptomic evidence that Rax-expressing cells in the organoid system are transitioning to the ocular fate. We found low levels of Rax expression in the GFP-negative samples on days 4 and 5 (Fig. 2A). This is to be expected as our cell-sorting strategy groups cells with low levels of GFP, including those just beginning to express Rax, into the GFP-negative samples.

In parallel with the upregulation of this extended set of putative eye-field genes, the cell-state transition was also characterized by the downregulation of a larger set of ‘EF-down’ genes (448) across the day 3-to-day 4/5 transition (Fig. S2A). Many of these genes are known players in pluripotency, including *Nanog*, *Pou5f1* and *Dppa5a* [more generally, 35 EF-down genes were linked to the gene ontology (GO) terms ‘mechanisms associated with pluripotency’ or ‘stem cell population maintenance’], and although they were downregulated across the state transition, they were also consistently downregulated across the full time course of organoid differentiation. This suggests that successful specification of the eye field requires both the network of eye-field genes to be turned on as well as genes controlling pluripotency to be switched off. It is noteworthy that *Sox2* and *Otx2*, known to be crucial in eye development, are not differentially expressed across this crucial time point (Fig. 2A), consistent with these genes being more broadly expressed in the anterior neuroectoderm *in vivo*. Furthermore, although Tcf family genes are important in repressing β-catenin/Wnt signalling to allow forebrain specification (Kim et al., 2000; Dorsky et al., 2003) and play a role in eye-field specification in zebrafish (Young et al., 2019), we found that key Tcf family genes, including *Tcf3*, *Tcf7*, *Tcf7l1* and *Tcf7l2*, are expressed in the OV organoids but not differentially expressed across this time point (Fig. 2A).

Next, we probed our RNA-seq data further to see whether any of the EF-up or EF-down genes displayed significant changes between earlier time points. Performing similar differential expression analyses, we found that nine (of 165) genes upregulated between days 2 and 3 overlapped with the EF-up genes, including the TFs *Rax*, *Lhx2*, *Six3* and *Fezf1*. The fact that these key factors are significantly upregulated so early in OV development (prior to detection of a distinct GFP signal), may point to these TFs acting as very early eye-field determining factors. Interestingly, 12 genes upregulated between days 2 and 3 actually overlapped with EF-down genes, including *Wnt3*, *Wnt8a*, *Fgf8* and *Lmx1b*, pointing towards the eye-field state requiring repression of gene expression programmes that were turned on earlier in differentiation. This has close parallels with what is thought to happen *in vivo*, where, as well as promoting ocular fate, EFTFs are also involved in repressing gene expression programmes characterizing other neural cell states, such as diencephalic, mid- and hindbrain states, early in development (Roy et al., 2013; Fish et al., 2014; Liu et al., 2010; Takata et al., 2017).

Finally, to explore whether there are general patterns to the trajectories of differentially expressed genes, we performed a clustering analysis on the trajectories of the EF-up and EF-down genes (Fig. 2B). This analysis resulted in six clusters, which fall into three trajectory patterns: genes in clusters 0 are steeply upregulated after day 3 (all the EF-up genes fell into this category); clusters 2, 3 and 5 contain genes with expression gradually downregulated with time; and clusters 1 and 4 contain genes with more transient expression, being broadly upregulated until day 3 and then sharply repressed after day 3. GO enrichment analysis (Zhou et al., 2019) on the gene sets displaying these three broad patterns provided an additional layer of evidence that the stable onset of the eye field is characterized by a balance of very different gene expression programmes: the upregulation of genes specifically related to eye and neuron development (Fig. S2D), and, in parallel, the downregulation of genes characterizing pluripotency, non-neuroectodermal differentiation (Fig. S2F) and possibly cell signalling and migration (Fig. S2E). In summary, RNA-seq data generated from the time course of OV organoid development show that this is a robust and relevant model system with which to study the gene dynamics underlying mammalian eye-field specification.

### Dynamic ATAC-seq peaks local to eye-field genes suggest enhancer-driven logic

Changes in the expression of genes characterizing cell-state transitions, in development and disease, are thought to be largely controlled by changes in DNA accessibility and binding of TFs at CREs genomically local to those genes (Spitz and Furlong, 2012; Ong and Corces, 2012; Buecker and Wysocka, 2012; Long et al., 2016; Klemm et al., 2019; Ray-Jones and Spivakov, 2021; Minnoye et al., 2021). To gain insight into the regulation of the transcriptional changes important for the eye-field transition, and specifically to identify candidate TF-CRE pairs that control the expression of EF genes, we performed bulk assay for transposase-accessible chromatin with sequencing (ATAC-seq; Buenrostro et al., 2013) experiments on single samples of organoids at days 0, 1, 2 and 3, as well as GFP-positive and GFP-negative cells on day 5. We identified a consensus set of 361,867 genomic regions displaying a significant accessibility peak on one or more days. Mirroring the strategy used in the RNA-seq analysis, we looked for peak regions that were dynamic (absolute log2-fold-change >1.5) across days 3 and 5, and found 7782 and 53 such peaks with increased and decreased ATAC-seq signal, respectively. After excluding peaks overlapping exons (TFs typically bind non-exonic regions), we found that this set of dynamic peaks comprises largely (∼97%) non-promoter peaks. This is consistent with previous work indicating that changes in the chromatin accessibility landscape during early development predominantly occur in non-promoter regions (Reddington et al., 2020), suggesting an enhancer-driven logic to cell-state changes.

We used GREAT analysis (McLean et al., 2010) to computationally assign putative links between these dynamic ATAC-seq peaks to genes in an unbiased manner. This revealed gene–peak associations for 22 of the 37 EF-up and 161 of the 448 EF-down genes, suggesting that the observed changes in chromatin accessibility may be functionally relevant in the regulation of genes characterizing the eye field.

**Fig. 3.**
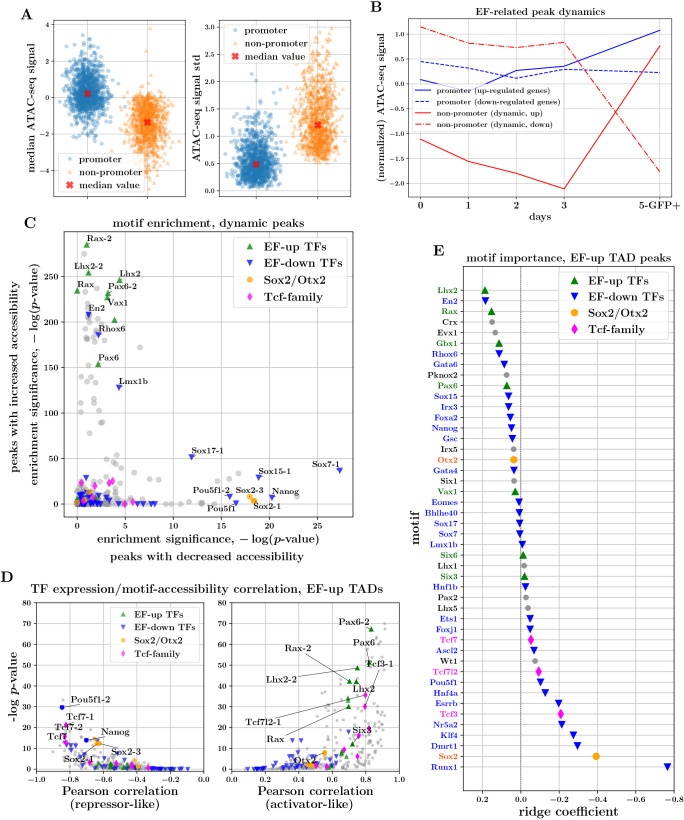
**Characteristics of ATAC-seq peaks associated with the transition to eye field point to enhancer-driven logic and regulatory roles for canonical EFTFs.** (A) Mean and variability of ATAC-seq signal in promoter and non-promoter peaks associated with EF genes. (B) ATAC-seq trajectories of EF-up and EF-down gene promoters, and dynamic non-promoter peaks within EF TADs. (C) TF-motif enrichment analysis of peaks with increasing (*y*-axis) and decreasing (*x*-axis) accessibility across the transition from day 3 to day 5. (D) TF-expression/TF-motif-accessibility correlations for peaks within EF-up TADs. Plots on the right and left illustrate the median across positive and negative correlations, respectively, indicative of activator-like and repressor-like behaviour of the respective TFs. (E) Coefficients of the logistic-regression model trained to predict opening versus closing ATAC-seq peaks within EF-up TADs, using the presence of TF motifs as input covariates. The magnitude of coefficients is indicative of the importance of the respective TF motifs for these predictions.

Our key goal was to uncover changes in chromatin accessibility that play a direct role in regulating the changes in gene expression identified using RNA-seq data. To identify CREs relevant to the putative eye-field genes, we focused on peaks that lie within the topologically associating domains (TADs) (Dixon et al., 2012; Nora et al., 2012) containing those genes (using TADs defined from previously published mESC Hi-C data; Bonev et al., 2017). This approach was motivated by the observation that TADs are remarkably stable across development and tissue types and that regulatory elements generally lie within the TAD containing the target gene (Symmons et al., 2016). We found that 98% and 93% of dynamic peaks in up- and downregulated eye-field TADs, respectively, correspond to non-promoter peaks. By contrast, peaks overlapping the promoters of many eye-field genes, including *Rax*, *Pax6* and *Lhx2*, were accessible from day 0, despite very low or zero corresponding gene expression (Fig. 3B; Fig. S3A). Indeed, whereas promoter regions of the putative eye-field genes displayed higher median ATAC-seq signals than the corresponding dynamic non-promoters (Fig. 3A; *P*<10^−229^, Mann–Whitney *U*-test), trajectories of the latter were more variable across the time course (Fig. 3A; *P*<10^−243^, Mann–Whitney *U*-test, and Fig. 3B). The above observations are suggestive of a model whereby gene-expression changes underlying the transition to ocular fate are orchestrated largely by changes in accessibility and TF binding at distal and intronic regulatory elements.

### Motifs of upregulated eye-field genes are enriched in and predictive of regions of dynamic chromatin

As TFs bind DNA in a sequence-biased manner, analysing the sequence content of accessible genomic regions provides preliminary evidence of the TFs that may be involved in gene regulation. We performed motif enrichment analysis on the peaks identified as dynamic across the transition from day 3 to day 5 (Fornes et al., 2020; Grant et al., 2011). We found that peaks with increasing accessibility have a strong enrichment for the motifs of the canonical EFTFs *Rax*, *Lhx2* and *Pax6*, whereas peaks with decreasing accessibility appear enriched for pluripotency factors motifs such as *Pou5f1* and *Nanog* (Fig. 3C). Furthermore, a *de novo* motif discovery analysis on peaks with increasing accessibility revealed that the inferred enriched motifs are most similar to (amongst others) the consensus motifs for *Rax*, *Lhx2* and *Pax6* (see Table S5). This indicates that TFs characterizing the transcriptomic state transition may also play an important role in re-shaping and/or activating regulatory regions of chromatin. To assess whether the evidence from the motif-enrichment analysis of genome-wide dynamic peaks is consistent with accessibility dynamics in peaks related to the EF-up genes, we performed a multivariate determination of motif importance for peaks within EF-up TADs. Specifically, we fitted a logistic-regression model to predict whether the accessibility signal of a peak increases or decreases across the transition from day 3 to day 5, using as input the binary motif presence of differentially expressed TFs. We found that the motifs corresponding to upregulated TFs, in particular *Lhx2* and *Rax*, are most predictive of opening chromatin, whereas motifs corresponding to pluripotency factors are predictive of closing chromatin (Fig. 3E). The overall picture of EFTFs and TFs associated with pluripotency being predictive of opening and closing chromatin peaks, respectively, appears to be a genome-wide pattern as we found very similar coefficient patterns when we repeated the modelling exercise for genome-wide dynamic peaks instead of peaks in EF-up TADs (Fig. S3B).

This multivariate approach also reveals that there are a number of downregulated TFs that are predictive of increases in accessibility. This could be explained by considering that a subset of the highest-scoring downregulated TFs, including *En2*, *Gata6* and *Irx3* have been reported in other biological contexts to act as transcriptional repressors (Tolkunova et al., 1998; Whissell et al., 2014; Bilioni et al., 2005) and therefore their downregulation may explain some of the increases in accessibility. A second possible explanation is that motifs for many TFs are similar and have overlapping occurrences across the genome (our motif discovery analysis showed that *En2* and *Lmx1b* motifs display significant similarities to the same *de novo* motif that strongly matches *Rax* and *Lhx2*; Table S5). This common issue with standard motif analyses makes it difficult to gain deeper insights into the molecular logic. One approach to move towards a more reliable set of regulatory TFs is to use ATAC-seq together with RNA-seq data. Assuming that a TF that alters the accessibility of regulatory elements must be expressed to do so, there ought to be a correlation between the accessibility of its DNA binding motif and its gene expression. Using an approach similar to that of Corces et al. (2016) and Berest et al. (2019), for each TF we computed the correlation between its expression and the accessibility of each peak containing the TF motif, and then compared the distributions of positive and negative correlations against relevant background distributions to determine significance. We found that for non-promoter peaks within EF-up gene TADs, the canonical EF genes *Rax*, *Pax6*, *Lhx2* and *Six3* as well as *Tcf3* and *Tcf7l2* displayed a significant positive expression-accessibility correlation (Fig. 3D; median corr >0.5, *P*<10^−10^ Wilcoxon rank-sum test), suggesting activator roles. The same analysis revealed potential repressive roles for the pluripotency factors *Pou5f1*, *Nanog* and *Sox2*, which displayed significant negative expression-accessibility correlation (Fig. 3D; median corr <− 0.5, *P*<10^−10^ Wilcoxon rank-sum test) for peaks within EF-up gene TADs. This is in line with evidence suggesting that, as well as being important for activating CREs maintaining pluripotency, these genes also act in a repressive manner to block activation of elements crucial for the upregulation of differentiation genes (Thomson et al., 2011; Wang et al., 2012). Similarly, we found evidence of a strong negative expression-accessibility correlation for *Tcf7* (Fig. 3D). Applying this analysis to peaks in EF-down TADs (Fig. S3C), we found that pluripotency factors there appear to have activator roles, in line with knowledge that these TFs bind enhancers that upregulate their expression. Surprisingly, we also found that peaks within EF-down TADs containing EFTF motifs tend to have an accessibility signal that correlates well with EFTF expression (Fig. S3C). This observation hints that, alongside being crucial for specifying the eye field, the EFTFs may also play a role at CREs repressing genes characteristic of alternative gene expression programmes (consistent with evidence that EFTFs repress alternative cell fates *in vivo*; Roy et al., 2013; Fish et al., 2014; Liu et al., 2010; Takata et al., 2017).

Taken together, the above TF-motif guided analyses and modelling of chromatin-accessibility changes point to the hypothesis that the stable upregulation of eye-field genes is enabled through the gain of binding of a small set of EFTFs at non-promoter CREs that are becoming increasingly accessible. Additionally, our analyses provide evidence that TFs downregulated across OV development act in a repressive manner. Therefore, loss of their binding at CREs with decreasing accessibility, perhaps through loss of ‘silencing’, may also contribute to the activation of the EF-up genes.

### Footprinting analysis provides evidence of differential binding of TFs

Detectable ‘footprints’ or relative depletions in chromatin accessibility signal at regulatory elements, can result from DNA being protected from transposase cleavage owing to the presence of bound TFs, and can provide indirect evidence of TF binding. Footprinting analyses have the potential to distinguish subtle effects, such as changes in the distribution of TF binding across conditions, which can be particularly useful if regulatory elements do not display overall changes in accessibility. We applied the TOBIAS framework (Bentsen et al., 2020) to our ATAC-seq data to identify putative changes in binding and footprinting of expressed TFs, both at a genome-wide level and in EF-TAD peaks. TOBIAS computes a ‘footprint score’ for each occurrence of a TF motif, which represents how well the underlying sequence matches the TF motif and the relative depletion in chromatin accessibility at the motif occurrence.

**Fig. 4.**
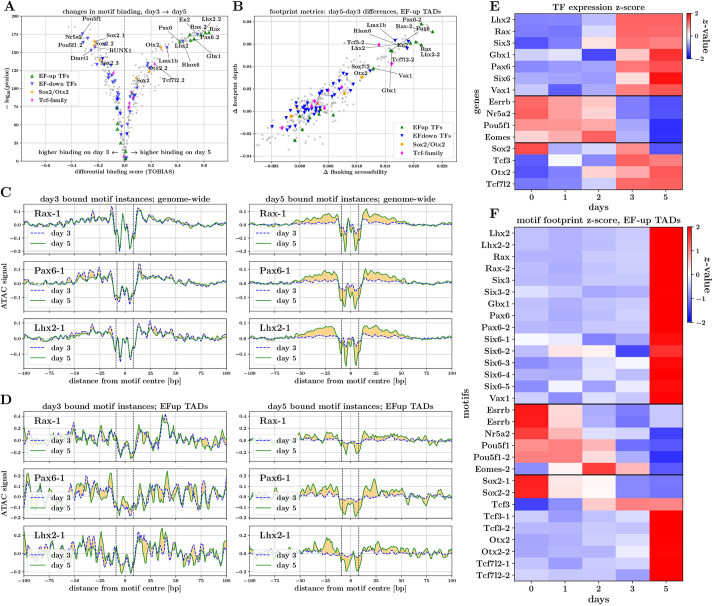
**Footprinting analysis provides evidence of direct interactions of EFTFs with DNA.** (A) Differential binding scores for days 3 and 5 for motifs of expressed TFs. (B) Scatterplot of day 3-day 5 differences in TF-motif footprint depth versus flanking accessibility, for peaks in EF-up TADs. (C,D) Aggregate footprint signals for *Rax*, *Pax6* and *Lhx2* motifs. Plots illustrate aggregate signal on day 3 and day 5 for motif instances predicted to be bound on day 3 (left) and day 5 (right). Aggregate footprint signals are shown for genome-wide (C) and EF-up TAD (D) motif instances. (E,F) Expression and motif footprint score trajectories for TFs displaying strongest expression-footprint correlations in EF-up TADs. (E) Heatmap of TF-expression values (average over triplicates) ***z***-transformed across the time course. (F) Heatmap of TF-motif footprint scores in EF-up TAD peaks, ***z***-transformed across the time course (median across peaks).

We performed differential binding analysis between days 3 and 5 to compare footprint scores for every TF-motif occurrence across the genome (providing an aggregate pattern across all occurrences of a motif within peaks of interest). This revealed that some of the largest increases in motif binding correspond to EF-up TFs, most notably Rax, Pax6 and Lhx2 motifs (Fig. 4A), and, similarly, that some of the most significant decreases in motif binding correspond to EF-down TFs, such as Pou5f1 and Dmrt1. Consistent with our motif analysis in the previous section, Six3 did not have a strong increase in binding across the transition from day 3 to day 5, perhaps indicating that Six3 plays a regulatory role in eye-field specification that does not involve direct binding to regulatory elements of EF genes (e.g. Six3-mediated Wnt8 suppression has been shown to be important for neuroretina development; Liu et al., 2010). Interestingly, we found evidence of increased binding of Otx2, consistent with the hypothesis that, together with Sox2, Otx2 is required for coordinated upregulation of the EFTFs and, in particular, leads to activation of *Rax* expression (Danno et al., 2008).

We assume that a TF playing a regulatory role by direct binding to DNA should display well-correlated footprint-score and expression trajectories. To investigate this, for each motif we computed the correlation of the footprint score of each occurrence with expression of the corresponding TF. The set of motifs that displayed a median correlation >0.5 in EF-up-TAD peaks contained all key EF-up TFs (Fig. 4E,F), plus Sox2, Otx2, Tcf3 and Tcf7l2. When comparing footprint score dynamics in EF-up-TAD peaks and genome wide, we found that there is generally good agreement for most motifs. Notably, however, footprint scores for Six3 and Six6 motifs broke this trend and genome-wide occurrences did not correlate well with their corresponding expression level (Fig. S4B).

Aggregating ATAC-seq signal around multiple occurrences of a given motif allows visualization and quantification of TF footprints. To unpick large changes in footprinting between days 3 and 5 in an unbiased manner, we computed the depth and flanking accessibility for each motif, for genome-wide and EF-up TAD peak occurrences, on each day. Comparing the changes in footprint depth and flanking accessibility between these time points, we found that in EF-up peaks (Fig. 4B) as well as genome wide (Fig. S4A), motifs of the upregulated TFs Rax, Pax6 and Lhx2 (as well as Vax1 and Gbx1) display some of the largest positive changes amongst all motifs of expressed TFs. Similar large increases in these scores were also found for the downregulated TFs Lmx1b, En2 and Rhox6, drawing parallels with our modelling of opening/closing chromatin peaks using motifs (Fig. 3E).

Finally, to confirm that the increases in footprint depth scores correspond to visually discernible footprints, we visualized the aggregate ATAC-seq signal at and around occurrences of a given motif. To pull apart changes between days, we compared the aggregate signal for motif occurrences that are predicted by TOBIAS to be ‘bound’ on day 3 and on day 5. Although there was little difference in aggregate signal between days, for occurrences predicted to be bound on day 3, we found a striking increase in footprint depths for Rax, Pax6 and Lhx2 motif occurrences predicted to be bound on day 5. This observation applied both to genome-wide peaks as well as to peaks contained in EF-up gene TADs (Fig. 4C,D). This provides further evidence that these TFs are regulating the onset of eye-field specification by binding elements in the genomic locality of EF-up genes. We also found that aggregate footprints for Pou5f1, Sox2 and Otx2 show differences (albeit less striking) that are consistent with eye-field establishment being correlated with loss of pluripotency factor binding and gain of Otx2 binding (Fig. S4C,D). Finally, the aggregate footprints for one of the Tcf3 motifs showed evidence of an increase in binding on day 5, both genome wide and within EF-up peaks (Fig. S4E,F), providing further hints at a direct regulatory role of this TF (or the Tcf family more generally) in eye-field specification.

### Peak and TF-specific footprint-score changes identify candidate *cis*-regulatory elements

The motif and footprint analyses described above provide consistent evidence that the canonical EFTFs Rax, Pax6 and Lhx2 play a direct regulatory role in establishing a stable eye field, specifically through binding non-promoter elements in accessible regions within TADs containing EF-up genes. Next, we moved from this broad picture of regulation towards identifying candidate gene-specific CREs through which these key TFs act. For a given EF-up gene, we approached this by searching for ATAC-seq peak regions within the TAD containing the gene, which displayed large changes in footprint scores for motifs of any of these three key TFs, as well as of *Sox2*, *Otx2*, *Tcf3*, *Tcf7* and *Tcf7l2*. We performed this focused intra-TAD analysis for the five core EFTFs (*Rax*, *Pax6*, *Lhx2*, *Six3* and *Six6*), looking for changes between days 1, 3 and 5 that either mirror the upregulation of the corresponding gene or which point to a loss of repression.

**Fig. 5.**
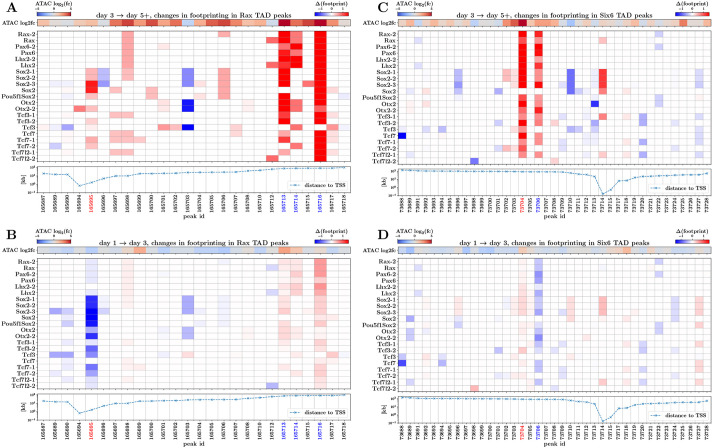
**Changes in motif footprint scores of key TFs identify candidate *Rax* and *Six6* CREs.** (A) Heatmap of changes between days 3 and 5 in footprint scores for key TF motifs at peaks in the *Rax* TAD region. Also illustrated are changes in ATAC-seq signal (top), and distance from the *Rax* TSS (bottom) for each peak. (B) As in A, but displaying changes between days 1 and 3. (C,D) As in A,B, but displaying changes in peaks contained in the *Six6* TAD. Red peak IDs indicate those chosen for CRISPR perturbation; blue peak IDs indicate other putative CREs with interesting footprint score changes (see also Fig. S5).

This approach is visualized in Fig. 5 for peaks within the *Rax* (Fig. 5A,B) and *Six6* (Fig. 5C,D) TADs, across the day 3-5 and day 1-3 time points. Focusing on changes from day 3 to day 5 within the *Rax* TAD (Fig. 5A), the heatmap revealed highly suggestive footprint-score changes across the majority of the key TFs, in three peak regions (peak IDs 165713, 165714 and 165716), mirroring upregulation of the *Rax* gene itself (Fig. S5A). A similar pattern was seen within the *Six6* TAD (Fig. 5C) where two peak regions (peak IDs 73704 and 73706) showed high footprint-score changes and increasing accessibility (Fig. S5B). No striking increases in footprint scores were identified across the day1-3 transition; however, there were peaks that showed a decrease in scores particularly for the Sox2-related motifs (most notably peak ID 165695 proximal to *Rax*). We found similar signal patterns, highlighting a small number of peaks for each EF-up gene (results for *Pax6*, *Lhx2* and *Six3* are shown in Fig. S6).

**Fig. 6.**
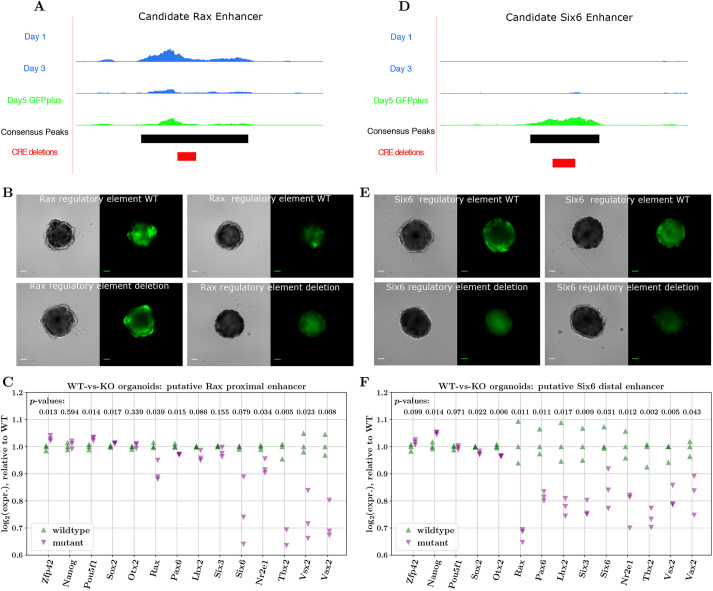
**Changes in expression of key TFs upon disruption of putative EFTF CREs.** (A,D) Bigwig tracks of candidate *Rax* and *Six6* enhancers, displaying accessibility changes across days 1, 3 and 5, consensus peaks and regions deleted by CRISPR. (B,E) Brightfield and GFP images of day 5 organoids derived from CRISPR-Cas9-edited cell lines with wild-type (WT; top) or mutant sequences for the predicted *Rax* and *Six6* CREs. Images represent the variability seen in organoid structure and GFP expression. Twenty-four organoids were grown in three replicates, and all showed similar levels of variation between organoids. Scale bars: 100 µm. (C,F) NanoString quantification of gene expression between WT and mutant (KO) organoids for the proximal *Rax* (C) and distal *Six6* (F) enhancers (WT/mutant differences in log2 expression tested using Welch's *t*-test).

As a step towards validating our approach for identifying gene-specific CREs, we selected two of the EFTFs for follow-up testing: *Rax*, because of the GFP readout of *Rax* expression within the organoid model system, and *Six6*, because of the relative simplicity of the intra-TAD footprinting patterns. We then selected one representative candidate CRE for *Rax* and one for *Six6* (Fig. 5, red peak IDs) to test for enhancer-like behaviour. Although accessibility of the candidate peak proximal to *Rax* remained relatively stable over time (Fig. 6A; Fig. S5A), the footprint-score changes indicate loss of Sox2 binding from day 1 to day 3 and then gain of binding from day 3 to day 5, suggesting this peak's function may switch from repressive to activating, a pattern not seen in other peaks in this TAD. This region was further prioritised as it overlaps with a putative CRE identified by Danno et al. (2008) in *Xenopus* (discussed further in the next section). The candidate peak selected for *Six6* was in a closed chromatin state at day 1, became accessible by day 5 (Fig. 6D; Fig. S5B) and had the largest increases in footprint scores from day 3 to day 5 (within the *Six6* TAD).

We used CRISPR-Cas9 (Ran et al., 2013) to introduce deletions encompassing the TF-binding motifs identified in the candidate CREs into the Rax-GFP cell line (see supplementary Materials and Methods). In the *Rax* peak, we introduced a 113-bp homozygous deletion (chr18:65941339-65941452) (Fig. S7A) that disrupts motifs for Sox2, Sox2-Pou5f1, Otx2, Pax6 and Tcf3. This includes the Sox2 and Otx2 motifs identified as regulating *Rax* expression in *Xenopus* (Danno et al., 2008). In the *Six6* peak, a 140-bp homozygous deletion (chr12:72852423-72852563) (Fig. S7B) disrupted one site predicted to be bound by Lhx2, Rax and Pax6, and additionally disrupts Sox2- and Tcf3-binding motifs. We used these cell lines to generate day 5 OV organoids and compared their differentiation with control cell lines that had been transfected with CRISPR-Cas9 reagents but retained the wild-type sequence (Fig. 6B,E). The organoids grown from all CRISPR-Cas9-transfected cell lines were considerably more variable in terms of the OV-like structures formed and the levels of GFP expression than previous batches of organoids, (Figs S8, S9), likely owing to the stress the cells were subjected to throughout the genome-editing process, meaning no clear conclusions could be drawn from imaging alone. To test the hypothesis that these genomic regions act as gene-specific CREs, and that perturbing them has a measurable effect on expression of the target gene, we performed digital gene expression (NanoString) analysis (Geiss et al., 2008) in triplicate on organoids at day 5 of differentiation, grown from cell lines that had no detected mutations introduced by the CRISPR machinery (referred to as wild type) and cell lines with the mutations described above.

Comparing the expression of key genes in wild-type and mutant organoids, we found that disrupting the candidate CREs for both *Rax* and *Six6*, results in downregulation of the associated target gene (Fig. 6C,F; − 11% for *Rax* and − 16% for *Six6* log2 expression, respectively). We highlight that developmental genes are generally regulated by more than one CRE (Buecker and Wysocka, 2012; Spitz and Furlong, 2012; Long et al., 2016), and therefore we do not expect complete ablation of *Rax* or *Six6* expression. Notably, for both mutants, *Tbx2* was one of the most downregulated genes, further supporting our hypothesis that *Tbx2* is the mammalian equivalent of *Tbx3* in *Xenopus*. Furthermore, we also found that in both *Rax* and *Six6* putative CRE mutant organoids, the expression of *all* EFTFs is reduced, as might be expected if this set of key TFs co-regulate each other (directly or indirectly) within a GRN. Interestingly, in line with the notion that the EFTFs play a role in suppression of the pluripotency programme, we found evidence of pluripotency genes (e.g. *Zfp42* and *Pou5f1*) being more highly expressed in mutant compared with wild-type organoids. Conversely, markers of later eye development (e.g. *Vsx2* and *Vax2*) were significantly downregulated in mutant organoids.

### Regulatory switches responding to ratios of TF concentration may trigger initial EFTF activation

With the resolution of our data, the mechanism actually triggering the expression of key EFTFs is difficult to disentangle. To answer this, we note that experiments (albeit in model systems different to mESC-derived organoids) in which just one of the TFs *Rax*, *Pax6*, *Lhx2* and *Six3* has been knocked out or rendered null, have shown that expression of the non-perturbed genes is activated in the appropriate regions (Zhang et al., 2000; Fish et al., 2014; Grindley et al., 1995; Bernier et al., 2001; Yun et al., 2009; Roy et al., 2013; Liu et al., 2010). Maintenance of this expression is observed to be significantly de-stabilized. This promotes the hypothesis that the events leading to onset of individual EFTF expression are not dependent on the EFTFs themselves.

These observations indicate that molecular events resulting in *Rax* being switched on, and specifically, the changes at CREs triggering *Rax* expression, do not necessarily involve other EFTFs (*Pax6*, *Lhx2*, *Six3*). Related to this, the work of Danno et al. (2008) showed, in *Xenopus* animal cap cells, that Sox2 and Otx2 proteins interact to activate *Rax* expression, by co-binding a conserved 35-bp region within a CRE ∼2 kb upstream of the *Rax* promoter. The authors propose that activation of this element is sensitive to the ratio of Sox2 and Otx2 concentrations (Fig. S10A).

As mentioned, the OV organoid ATAC-seq data we have generated in this study contains a peak that overlaps with the conserved element identified by Danno et al. (2008). Intriguingly, we find that this putative CRE is accessible (with no large changes in overall accessibility) throughout the organoid development time course, and thus appears to be primed for activation. Furthermore, if it is the ratio of Sox2 and Otx2 that leads to the activation of this CRE, then we would expect to see sizeable differences in Sox2 and Otx2 binding between OV development days. Indeed, this is what we did find for Sox2; the footprint score for Sox2 motifs decreased from day 1 to day 3 and increased again from day 3 to day 5, specifically at this regulatory element, possibly indicating changing of Sox2 binding partners across the time course (Fig. 5A,B). The predicted binding ratios for Otx2 at this element were less pronounced, although they did increase from day 3 to day 5, in line with the working hypothesis.

To gain further insights into the activation of this element, we mined the UniBind database (Puig et al., 2021) of TF–DNA interactions for robust (combined ChIP-seq and computational modelling) evidence of binding sites of specific TFs in this genomic region, across all (mouse) experimental models. This revealed several TF-binding sites that may be relevant to our system (Fig. S10B), including sites for Sox2 and Otx2 in close proximity. Interestingly, the Otx2 site proximal to the Sox2 site overlaps a UniBind Pou5f1 TF-binding site, which, taken with the corresponding expression of these TFs and our footprint scores, provide further suggestion that swapping of Sox2 binding partners is a plausible hypothesis for the activation of this regulatory element. Furthermore, we report that by exploring two publicly available Sox2 and Pou5f1 ChIP-seq datasets (Xiong et al., 2022; Avsec et al., 2021) generated from E14-mESCs (roughly equivalent to day 0 organoids), we found peaks of Pou5f1 and Sox2 signal at this CRE (Fig. S10C). In all, this suggests a mechanism whereby, although this element is accessible early in development, it is repressed by Pou5f1 binding and thus not able to activate *Rax* expression. As the expression of *Pou5f1* is downregulated and the expression of *Otx2* increases, Sox2 can exchange binding partners (from Pou5f1 to Otx2) and, in so doing, activate this putative enhancer and consequently also the expression of *Rax*.

Further experiments (in particular a CHIP-seq or Cut&Run time course for the relevant TFs) beyond the scope of our current study would be required to validate this hypothesis. We do, however, find it both fascinating and of considerable value that specific hypotheses of gene regulation can be pieced together through systematic and integrated analysis of data across modalities, such as the one presented above. We also point out that, similar to the mechanics of regulation we hypothesize above, Delás et al. (2023) have recently provided evidence of a general mechanism whereby groups of genes are regulated via ‘differential binding’ of TFs at regulatory elements (rather than differential accessibility of these elements), in the context of neural progenitor differentiation.

## DISCUSSION

By using an OV organoid model to enrich for cells transitioning to an optic fate, we have generated bulk transcriptomic and chromatin accessibility time-course data from a very early developmental event that is crucial for mammalian eye development. Joint computational analyses of these data have revealed three key messages regarding the molecular events controlling eye field specification. First, the transition to the eye field in mESC-derived OV organoids is characterized by the upregulation of a small set of genes, including a core set of TFs known to be necessary for eye development through studies in *Xenopus*. Second, the patterns extracted from our RNA-seq data indicate that when this state transition occurs, not only is this core set of EFTFs upregulated, but, in parallel, other gene expression programmes, such as those controlling cellular pluripotency and cellular signalling, are downregulated or carefully balanced. Third, analysis of the dynamics and sequence composition of chromatin accessibility peaks strongly suggests that the key EFTFs – particularly *Rax*, *Pax6* and *Lhx2* – act to control the upregulation of the EF genes through binding to (primarily) non-promoter regulatory elements in the vicinity (TADs) of those genes. By performing footprinting analyses for key TFs in TADs of important genes, we have additionally identified putative gene-specific CREs that appear to be important for regulating the gene expression changes characterizing the onset of the eye field. Furthermore, by perturbing a subset of these candidate regulatory elements using CRISPR and generating mutant cell lines, we have started to validate the regulatory potential of these elements in controlling EFTF expression in OV organoids.

In all, our study complements the early characterization of the eye field from *Xenopus*, extends this characterization to mammalian models and begins to shed light on the roles of key EFTFs in the transition of cells to the ocular fate. Additionally, our work demonstrates the power and practicality of using an organoid system to generate and test specific hypotheses regarding the regulation of cell-state transitions relevant to crucial events in mammalian development.

Although our data does not have the resolution to identify definitively what may initially trigger the expression of individual EFTFs, we have presented evidence to suggest that this may arise, at least in the case of *Rax*, from the replacement of repressive factors (such as Pou5f1) with activating factors (such as Otx2) at regulatory elements that are ubiquitously open across the time course. We have also outlined that disentangling the mechanisms behind gene-expression or cellular-state changes that are triggered through the action of TFs at regulatory elements can indeed be very challenging to understand through standard analyses correlating peak accessibility with gene expression. However, footprinting analyses do have the potential to provide richer insights into these more subtle mechanisms of regulation.

The approach to characterize the OV-organoid eye field presented in this work does have some limitations, which indicate areas for future work. First, at each time point the organoids are likely to be composed of relatively heterogeneous collections of cells. As a result, the bulk RNA-seq and ATAC-seq datasets generated for this study are expected to be powerful enough to reveal the larger changes in gene expression programmes, such as the upregulation of the eye field programme or the suppression of pluripotency, but may be limited in their power to distinguish more subtle effects. To address the confounding issues introduced by cellular heterogeneity, an approach profiling the organoids at the single-cell level would be required. Second, although motif and footprinting analyses of our ATAC-seq data provide consistent evidence to build hypotheses regarding the putative regulatory roles and binding of important TFs, this evidence is indirect (the occurrence of a consensus TF motif within a region of enriched accessibility does not imply the binding of that TF at that genomic location). To gain more direct evidence of changes in TF binding it would be useful to perform time-course ChIP-seq or Cut&Run experiments for a set of TFs identified as important.

Finally, in undertaking the work presented here we have encountered a number of intriguing open questions regarding eye-field specification that require further study. In particular, it is pertinent to ask whether the eye field directly stems, in a sequential manner, from a group of well-defined progenitor cells within the neural plate, or whether individual neuroectodermal cells spontaneously acquire ocular fate and are only then organized into a coherent eye field through migration and cell-signalling events? Furthermore, are these processes similar *in vivo* and in the organoid system? Using a collection of markers Agnès et al. (2022) have recently quantified, in 3D, the emergence of the eye field in dimorphic teleosts and it would be informative to gain a similar spatial molecular map of the eye field in mammalian systems. Related to our observations that eye-field specification seems to require a careful balance of different, potentially competing gene expression programmes, an interesting avenue of further study would be to quantify the effects that perturbations of gene dosage have on this balance (particularly as dosage of key genes has been shown to affect stability of the eye field; Beccari et al., 2012; Bernard et al., 2014). To probe further the emergence of the eye field, it would be fascinating to investigate the extent to which the EFTFs act in a cell-autonomous manner, regulating the genes of the cells in which they are endogenously produced, or whether there are non-cell-autonomous components to gene regulation (paracrine signalling). Recent rapid technological progress in single-cell technologies – both multi-omics and imaging – will enable exciting future research in these directions. Finally, in order to bring this research closer to having translational potential, similar analyses must be performed using organoids derived from human ESCs; indeed, such a line of work has the potential to identify and test mechanisms crucial to early human eye development and disease that are currently impossible to study.

## MATERIALS AND METHODS

### ESC maintenance and organoid culture

Rx-GFP mouse ESCs were maintained and SFEBq (serum-free floating culture of embryoid body-like aggregates with quick aggregation) differentiation of OV organoids was performed as described previously (Wataya et al., 2008; Eiraku et al., 2011). Briefly, mESCs were dissociated and 4500 cells were plated in differentiation media [GMEM supplemented with 1.5% KOSR (KSR media) or a growth factor-free chemically defined medium (CDM media) for knockout organoids and initial organoids, respectively] in a low cell adhesion 96-well plate. This was defined as day 0. On day 1, growth factor-reduced Matrigel was added to a final concentration of 2%. For further details, see supplementary Materials and Methods.

Differentiation status was assessed using fluorescence imaging to detect the presence of GFP as a measure of *Rax* expression.

### RNA-seq assays

On days 0-5 of optic vesicle differentiation, triplicate samples of cells were collected from 24 organoids, which were dissociated and sorted using FACS to select for live single cells. Samples at days 4 and 5 were also sorted on the basis of GFP expression giving a GFP-positive and -negative sample for each of these time points. The FACS gating placement was designed to maximize the number of cells in each sample, such that some cells with low GFP signal were included in the GFP-negative sample, and the GFP-positive samples contained cells with both high and moderate levels of GFP expression. Day 3 samples were analysed with FACS and found to contain a small proportion of GFP-expressing cells, but not enough to extract sufficient RNA. To obtain a more accurate view of the diverging cell types at this stage, all live cells from these organoids, regardless of their GFP expression were included in this sample. RNA was isolated using the Zymo Direct-zol RNA MicroPrep kit following the manufacturer's instructions. Quality and integrity of the RNA samples were analysed, libraries were prepared using the TruSeq kit (Illumina), and sequenced on the NextSeq 550 platform as per the manufacturer's protocols. For further details, see supplementary Materials and Methods.

### RNA-seq analysis

#### Mapping reads and quantification

Coding regions of DNA and non-coding RNA from the mouse genome build 38 (mm10), downloaded from the Ensembl database, were used to create the transcriptome index. kallisto (Bray et al., 2016) was then used to pseudo-align RNA-seq reads for each library to these regions and quantify transcript abundance.

#### Differential expression analysis

Differential expression analysis between time points and expression normalization was performed using DESeq2 (Love et al., 2014). Specifically, the kallisto abundance estimates were used as inputs to DESeq2, using a design matrix of time points. The ‘estimateSizeFactors’ function was used to compute size factors for normalization, and the ‘lfcShrink’ function, with type=‘ashr’ for log2-fold-change shrinkage (Stephens, 2016), was used to contrast time points. To select differentially expressed genes, we used an FDR threshold of 0.001 and an (absolute) log2-fold-change threshold of 1.5. To select genes of particular relevance to the transition between day 3 cells and cells on the RaxGFP-positive lineage (days 4 and 5), we performed differential expression analysis for the time points day 3/day 4GFP^+^, day 4GFP^−^/day 4GFP^+^, day 5GFP^−^/day 5GFP^+^. Genes found to be significantly upregulated across all these comparisons were termed ‘EF-up’ genes, whereas those significantly downregulated across these comparisons were termed ‘EF-down’ genes. We also used DEseq2 to select a set of ‘housekeeping’ or stably expressed genes, by requiring these genes to display absolute log2-fold-change <0.1 for every successive day comparison, and have a mean (across days) normalized expression >30 (this procedure identified 190 such stably expressed genes).

#### Clustering

To cluster gene expression trajectories, we used a Gaussian-mixture model (GMM) approach applied to *z*-transformed log2 values of normalized gene expression for EF-up and EF-down genes. In detail, we used the GMM implementation available in the Python scikit-learn library (Pedregosa et al., 2011), fitting GMMs on the trajectories for 20 random initializations. The number of clusters that best described the data was determined using the minimum value of the average Bayesian Information Criterion (BIC) across random seeds, and the cluster allocation of each gene was achieved by consensus clustering, using the ClusterEnsembles Python library.

#### GO enrichment analysis

We applied a GO enrichment analysis on the three sets of genes corresponding to the broad patterns discovered using clustering, using the Metascape online tool (Zhou et al., 2019; https://metascape.org).

### ATAC-seq assays

On days 0-5 of optic vesicle differentiation, cells from 48 organoids were pooled and sorted using FACS, as described above for the RNA-seq. One replicate was generated for each time point. ATAC-seq sample preparation was performed as described by Buenrostro et al. (2015). Briefly, 50,000 cells were lysed, and the transposition reaction carried out using the Tn5 transposase enzyme. PCR amplification of samples was conducted using a universal forward primer and a unique barcoded reverse primer for each time point. Samples were then purified and size selected. Bioanalysis showed that the libraries contained the expected fragment size distribution (Buenrostro et al., 2013). ATAC-seq samples were paired-end sequenced on the Illumina Hi-seq platform, with day 4 samples run separately from the other time points. For further details, see supplementary Materials and Methods.

### ATAC-seq analysis

#### Mapping reads, quantification and peak-calling

We used the FastQC tool (http://www.bioinformatics.babraham.ac.uk/projects/fastqc/) and cutadapt (Martin, 2011) to perform quality control on and trim adapter sequences from the raw sequencing reads for all samples. Then we used bowtie2 (Langmead and Salzberg, 2012) to align trimmed reads to the mm10 genome. Duplicate reads, unmapped reads, and reads mapping to the mitochondrial genome were removed using samtools (Danecek et al., 2021). Filtered reads were shifted + 4 bp for the positive strand and − 5 bp for the negative strand to show the centre of the transposition site. bedtools (Quinlan and Hall, 2010) was used to generate the final bam and bed files used for downstream analysis. To enrich for fragments indicative of binding of TFs, we filtered out reads corresponding to fragment sizes >100 bp (i.e. retained only sub-nucleosomal reads for downstream analysis). To identify genomic regions enriched for accessible chromatin, we computationally called peaks using the callpeak function of macs2 (Zhang et al., 2008; https://github.com/taoliu/MACS), with parameters -f BAMPE -g mm –keep-dup all -B –SPMR –nomodel, on paired-end bam files of each sample (day) separately. Using the narrowPeak macs2 peak coordinates, we created a consensus set of peaks with which to study accessibility dynamics across days, using the bedtools merge command. From this consensus set of peaks, we removed peaks overlapping blacklisted regions of the mm10 genome, as listed on http://mitra.stanford.edu/kundaje/akundaje/release/blacklists/. We used conditional quantile normalization (Hansen et al., 2012) to normalize the signal in consensus ATAC-seq peaks while correcting for the effects of peak width and GC content. Owing to the separate sequencing runs, day 4 samples showed considerable batch effects and were removed from subsequent analysis. Bedgraph files from macs2 were converted to BigWig files for visualization in the UCSC genome browser. Finally, we used ChIPseeker (Yu et al., 2015) to annotate peaks by their genomic context, collapsing peak annotations into four categories: promoter, intergenic, intronic and exonic.

#### Peaks by TAD regions

To assign peaks to TAD regions, we downloaded the coordinates for TAD regions from the work of Bonev et al. (2017; publicly available at https://github.com/aertslab/mucistarget/tree/master/data/tads). We noted that there are often gaps between genomically adjacent TAD regions, and in these cases we took these inter-TAD regions to also define relevant regions to restrict the search-space of putative peak-gene links (interestingly, for the TAD data we used, the *Rax* and *Six6* genes were contained within such inter-TAD regions). EF-up and EF-down TADs were defined as those TADs containing the genes determined to be differentially up- and downregulated, respectively, across the transition from day 3 to day 5. Housekeeping TADs were defined as TAD regions containing genes that are stably expressed across the full time course.

#### GREAT analysis

To perform a first putative linking of dynamic peaks to genes we employed the online GREAT tool (McLean et al., 2010; http://great.stanford.edu) to generate region–gene associations computationally. We used the coordinates of dynamic peak regions (regions of increasing and decreasing accessibility across the transition from day 3 to day 5) as test regions, and the whole genome as the background regions (default setting of GREAT).

#### Motif analysis of ATAC-seq dynamics

We obtained the DNA sequence of each consensus peak using bedtools getfasta and the mm10 genome. Following this, we used fimo with parameters –text –max-strand –thresh 1e-4 (i.e. a *P*-value threshold of 1*e*^−4^), and we scanned each peak region for occurrences of all motifs in the JASPAR2020 database (*Mus musculus* TF motifs), using the presence/absence of motifs for further downstream analysis. We also restricted our attention to motifs of TFs that were expressed in the OV system, which we defined to be the mean (across RNA-seq replicates) DEseq2-normalized counts of a TF's expression >50, at any point in the time course. To compute enrichment of motifs for our defined signal peak sets [peaks with increasing or decreasing accessibility signal above a log2-fold-change of 1.5], we employed the hypergeometric test (Fisher test) using the set of peaks in TADs of housekeeping genes as a background.

Finally, to quantify the importance of each motif in a multivariate modelling approach, we fitted an *L*2-regularized logistic regression model to predict the binary behaviour of each peak (increasing or decreasing accessibility signal) using the presence of motifs corresponding to EF-up genes, EF-down genes, day2-day3 differentially expressed genes, as well as Sox2 and Otx2, as input features. Where there were multiple motifs for a TF, we collapsed the input feature into a single feature in which a TF motif was deemed to be present in a peak if that peak contained a significant occurrence of any of its possible motifs. We trained models on two sets of peaks: dynamic peaks [those with log2-fold-change >1] within TADs containing EF-up genes and genome-wide dynamic peaks. We first used 5-fold cross-validation to determine an appropriate regularization parameter for each model, and then trained models using these optimal parameters on the full sets of peaks. In this second step, we fitted ten models with different random initial seeds, and took the mean for each coefficient across each run. To perform this modelling, we used the ‘LogisticRegression’ functions implemented in the sklearn Python library (Pedregosa et al., 2011).

We performed our *de novo* motif discovery analysis using streme (Bailey, 2021) and matched the inferred motifs to the consensus ones in the JASPAR2020 database using tomtom (Gupta et al., 2007). Results of these analyses applied on peak regions increasing and decreasing in accessibility across the transition from day 3 to day 5 can be found in Tables S5 and S6.

ChIP-seq evidence-backed sites of TF–DNA interactions were obtained from the UniBind database, (Puig et al., 2021; https://unibind.uio.no/), and specifically downloading data from the *Mus musculus* genome tracks.

#### Footprinting analysis

To quantify differences in putative TF binding between time points, we used the TOBIAS framework (Bentsen et al., 2020). We first used the bam files (with subnucleosomal reads) from each time point (days 0, 1, 2, 3 and 5) as input to TOBIAS-ATACorrect to correct for Tn5-insertion bias, resulting in ‘corrected’ bigwig files of ATAC-seq signal. Together with the coordinates for the consensus peak set, the latter were used as input to TOBIAS-ScoreBigwig to compute base-pair footprint scores for these genomic regions, again in bigwig format. Finally, these footprint bigwig files were used as input to TOBIAS-BINDetect (in time-series mode, using the –time-series flag) to identify motif occurrences in the consensus peak set and compute associated footprint scores for each day, for all mouse motifs in the JASPAR2020 database (Fornes et al., 2020). This final step also quantified differences in putative binding of TFs across the full consensus peak set between each pair of successive days (used to produce the results of Fig. 4A). To generate the data for aggregate footprints (used for Fig. 4B and Fig. S4B), we used TOBIAS-PlotAggregate to average corrected ATAC-seq signal in ± 100 bp windows around occurrences of a given motif.

To quantify changes in aggregate footprints for a given TF motif, we first computed two metrics, ‘flanking accessibility’ and ‘footprint depth’ (closely following the approach of Baek et al., 2017), on the aggregate signal at each time point. For each motif, we took the ± 12 bp window around the motif centre as the ‘central region’ (putative region of binding), and first computed flanking accessibility as the mean aggregate signal in the 60 bp either side of the central region. Footprint depth was then computed as the mean aggregate signal within the 24-bp central region minus the flanking accessibility. Finally, we computed the difference between time points of each of these metrics to obtain the change in flanking accessibility and of footprint depth.

### CRISPR-Cas9-mediated disruption of candidate CREs

Mutations in the candidate regulatory elements within the Rax and Six6 TADs were introduced into the Rax-GFP cell line using CRISPR-Cas9. Guide RNAs, targeting the sites of TF-binding motifs within the identified peaks, were individually cloned into the pSpCas9(BB)-2A-GFP (PX458, Addgene plasmid #48138) vector. Rx-GFP cells were transfected with the resulting plasmid using Lipofectamine 3000 and cells selected based on GFP expression from the plasmid 48 h later. Cells were plated at low density, individual clones were expanded and resulting cell lines were assessed for deletions over the CREs in question using Sanger sequencing (Fig. S7). One cell line with a deletion encompassing motifs of interest along with one cell line without any deletions were maintained and used for optic vesicle organoid culture for each of the two selected putative CREs. At day 5, triplicate samples of 24 organoids were pooled, and RNA isolated as described for the RNA-seq assay. The NanoString nCounter analysis system (Geiss et al., 2008) was used to quantify RNA expression for a panel of 180 genes for which expression levels changed through the time course of differentiation. For further details, see supplementary Materials and Methods.

### Plotting and visualization

Plots and visualizations were produced using the matplotlib Python (Hunter, 2007) library and Inkscape (https://inkscape.org).

## Supplementary Material

10.1242/develop.201432_sup1 Supplementary information
