# Supplementary material for: Characterization of an eye field-like state during optic vesicle organoid development
Source: Development. 2023 Aug 9;150(15):dev201432. doi: 10.1242/dev.201432 (PMC10445745; doi:10.1242/dev.201432)
Supplement: Supplementary file 1 [file develop-150-201432_review_history.pdf]

Characterization of an eye field-like state during optic vesicle organoid development

Liusaidh J. Owen, Jacqueline Rainger, Hemant Bengani, Fiona Kilanowski, David R. FitzPatrick and Andrew S. Papanastasiou
DOI: 10.1242/dev.201432

Editor: Steve Wilson

Review timeline

Submission to Review Commons:	22 August 2022
Submission to Development:	7 November 2022
Editorial Decision:	28 November 2022
First revision received:	28 February 2023
Editorial decision:	11 April 2023
Second revision received:	30 May 2023
Accepted:	2 June 2023

Reviewer 1

Evidence, reproducibility and clarity

Summary

Owen et al. characterize the transcriptome and chromatin accessibility of mouse retinal organoids at early stages during which eye field-like cells are specified. Since cell specification and differentiation in retinal organoids largely mimic those processes in vivo, retinal organoids are viable models for studying the mechanisms of early eye development. Owen et al. utilize a previously established Rx-GFP cell line, bulk RNA sequencing, and bulk ATAC sequencing to dissect the mechanisms of early eye development in mice. Their findings are generally consistent with previous studies. Overall, the study is interesting for the field, but its conceptual and technical advances are moderate. In addition, a few major points need to be clarified.

Major points

1. The authors did not show any analysis of retinal organoids at stages when Vsx2 is expressed. This is a significant weakness since the chemically defined medium (CDM) used in Owen et al.'s study was previously shown to induce rostral hypothalamic differentiation (Wataya et al., 2008). Related to this notion, several eye-field transcription factors, such as Rax and Six3, are also expressed in the hypothalamus. Therefore, Owen et al. need to demonstrate that organoids in their modified differentiation system efficiently produce Vsx2- positive retinal progenitors, and samples of organoids at stages when Vsx2 is expressed should be included for RNA sequencing. If Vsx2 is not efficiently expressed in their organoids, the interpretation of results will be very different.

2. The authors state that "two differentiation medias were used for this work due to the differentiation becoming unstable after the initial experiments had been performed. The organoids used for RNA and ATAC-seq were grown in CDM media and the organoids with mutations introduced in potential CREs were grown in KSR media". Why the differentiation becomes unstable after the initial experiments? Differences in the two media cause additional complexities. Related to this notion, "WT Rx-GFP" in Figure 4B and 4E appears to show a different expression pattern compared to that in Figure 1A.

© 2023. Published by The Company of Biologists under the terms of the Creative Commons Attribution License (https://creativecommons.org/licenses/by/4.0/).

3. Is the deletion of Rax and Six6 regulatory elements homozygous? Sanger sequencing or amplicon sequencing is needed to show the deletion.

4. The deletion of Rax and Six6 regulatory elements appears to cause minor changes in the expression of Rax and Six6 (Figure 6C, F). Therefore, the impact of findings in bulk RNA seq and bulk ATAC seq in this study is still unclear.

5. Retinal organoids and sorted cells are composed of heterogeneous cell populations. Bulk RNA seq and bulk ATAC seq do not have the power to dissect the complexity of heterogeneous cell populations. Single-cell RNA seq and single-cell ATAC seq are more powerful for this study.

6. Numerous motifs in the JASPAR database are identified using in vitro assays and have not been validated using in vivo assays. Unexpected results in motif analysis could be due to the differences in DNA binding motifs between in vitro and in vivo conditions. This notion should be added in the discussion.

Minor points

Numerous labels in figures are too small.

Referees cross-commenting

My fellow reviewers identify similar major weaknesses and additional points. I agree with the other reviewers' comments.

Significance

Nature and Significance of the advances

In Owen et al.'s study, the Rx-GFP cell line and retinal differentiation protocol were established in previous studies (Wataya et al., 2008; Eiraku et al., 2011); bulk RNA sequencing and bulk ATAC sequencing are standard procedures. Although candidate regulatory elements for early eye development are identified, deletions of two prioritized elements using CRISPR/Cas9 only cause minor changes in the expression of targeted genes. Overall, conceptual and technical advances in Owen et al.'s study are moderate.

Compare to existing published knowledge

The datasets could be useful for the field, but conceptual and technical advances are moderate.

Audience

Developmental biologists, stem cell biologists, vision researchers.

Your expertise

Developmental biology, stem cell biology, vision research

Reviewer 2

Evidence, reproducibility and clarity
The authors grow eye organoids from cells with a reporter driving GFP in the Rax locus, a gene that is expressed in the eye field in many animal model systems. They show that expression of GFP picks up by day 4 and performed FACS sorting of GFP+ cells on day 4 and day 5 organoids to compare gene expression by RNAseq comparing with earlier day organoids. The data shows 37 genes with a differential expression on days 4 and 5, compared to day 3, and enriched in GFP+ cells, which they define as EF-up genes. It is notable that some of these genes had already been

© 2023. Published by The Company of Biologists under the terms of the Creative Commons Attribution License (https://creativecommons.org/licenses/by/4.0/).

identified as canonical eye field gene regulatory network transcription factors. In the same way, they identify a group of differentially expressed regulated genes, EF-down, and state that 'many' of them are involved in pluripotency. However, they do not mention how many, or the proportion of these genes in the whole list. It would be useful if they could provide the number to understand how many of these genes are related to pluripotency, the whole list of genes mentioned to be downregulated in a supplementary file. The authors also note that genes known to be required for eye specification like Sox2 and Otx2 are not differentially expressed across the day 3-4 timepoint (Ln 190). However, this is not surprising considering that both genes are broadly expressed in the anterior neural ectoderm and required for its specification, which should be noted by the authors.

The authors then go on and cluster the EF-up, EF-down and genes deferentially expressed between days 2 and 3, and identify 6 discreet trajectory groups. From this analysis, they identify a third group of genes which shows a peak on day 3 but whose expression falls on days 4 and 5. It is interesting to see that this group includes Wnt and Fgf morphogenes. The authors should provide a list of the genes in the different clusters for the readers to inspect and analyse. Aiming to generate insight into the cis-regulatory elements that regulate of the genes the authors found differentially expressed in their model system they performed a series of ATAC-seq experiments. When linking the genomic regions with differential ATAC-seq accessibility to gene locus using the GREAT analysis, they identified association to 22 of the EF-up and 161 of the EF-down genes. This suggests a functional link between the ATAC-seq genomic regions and the gene regulation of the differentially expressed genes.

The authors later screened the ATAC-seq regions of increased accessibility for TF binding motifs and found that these regions were enriched with motifs for EFTF gens Rax, Lhx2 and Pax6. When assessing motifs in the ATAC-seq regions in EF-up TADs, Rax and Lhx2 motifs scored highly associated to open chromatin positions. Authors also observe a positive gene expression-accessibility correlation between in Pax6, Lhx2, Six3 and Otx2, and suggest this could mean these genes activate transcription of the EF-up group of genes. The same analysis, but focusing on EF-down genes, suggests that EFTFs repress the expression of EF-down genes which include those involved in pluripotency.

Further interrogating the ATAC-seq data, the authors use TOBIAS footprinting analysis to identify changes in TF binging in EF-TADs and EF-up motifs. Remarkably, whole genome analysis reveals that the largest increase in motif binging corresponds to EF-up genes Rax, Pax6 and Lhx2. The authors then narrow down on specific gene regulation by studying the ATAC-seq data within the TAD of Rax and Six6. However, they do not explain the rationale for which these two genes were highlighted, and why Pax6 or Lhx2 were excluded. This explanation should be added to the manuscript. The analysis identifies three regulatory elements in the Rax TAD and two for Six6. They then go on and study one putative regulatory element of each gene and generate CRISPR deletions in cell lines. The rationale for the choice of these particular elements is not clear, nor if the cell lines are the same used for the RNAseq experiments. This information should be explicit in the results and in the methods section. The authors mention that the CRISPR cell lines are "considerably more variable" (Ln 822) compared to the previously studied organoids and suggest that no conclusions can be driven from GFP expression or morphology alone. However, they do not specify which is the variable trait. This information should be added to the text. The authors also miss out on specifying the time stage of the organoids in figure 6 which should be stated. Regardless, the wildtype organoids in figure 6 and figure S7 show a very different morphology and GFP expression compared to those in figure 1, suggesting that the conclusions from this last set of experiments are not reliable or comparable to those in figure

1. This, together with the fact that different reagents were used to grow the organoids for the RNAseq and the CRISPR experiments, is a weakness of this work that must be addressed.

The last part of the results section belongs to the discussion as no results generated by the researchers are included. The discussion in this paper is a good opportunity to state the limitation of this study.

© 2023. Published by The Company of Biologists under the terms of the Creative Commons Attribution License (https://creativecommons.org/licenses/by/4.0/).

Major comments to address

1. One of the main issues identified is that the morphology of the control conditions in the CRISPR experiments (Fig.6) do not look is that those used for the RNAseq experiments (Fig.1) and the authors should address this issue. The fact that CDM media was used on the RNA extraction and ATACseq experiments and then KSR media was used for the CRISPR experiments is worrying and makes one wonder whether the second set of experiments is at all comparable to the first. This should be somehow controlled carefully by at least replicating one set of RNA experiments with the KSR media.

2. The requirement of Wnt signalling inhibition has been well established as a requirement for forebrain specification, including the eye field. Considering the link of the Wnt/beta- catenin pathway to eye specification and that TCFs, the transcription factors that mediate Wnt pathway transcription regulation, have known and well-studied DNA motifs, it is surprising that authors do not include the analysis of TCF motifs in their study. Also considering that TCF7l1 (TCF3, old nomenclature) has recently been shown to be cell- autonomously required for the expression of rx3 (Rax homologue) in zebrafish. One would expect TCFs to be included in the analysis as it was done with Sox2 and Otx2, which were studied due to the known relevance in forebrain specification rather than from the direct analysis of the differential gene expression experiments.

Minor comments to address

1. The authors should clearly state the day timepoint used in the organoids experiments in the results section and figure legends, not just in the methods.

2. The report by Agnes et al Development 2022 should be cited in the introduction as it is an excellent paper related to this topic, including a comprehensive analysis of the EFTFs expression pattern.

3. Ln 41. Mutations in these genes do not always cause severe bilateral eye malformations. Probably best to moderate and mention that they 'can' cause these malformations.

4. Ln 146. Authors mention that in vitro organoid systems "closely mimic the in vitro regulatory dynamics". This statement should be moderated as we do not know if this is true. In fact, one of the positive aspects of this study is that it contributes to supporting this statement.

5. Ln 150. Rax homologue Rx3 is also expressed in cells that give rise to the hypothalamus in zebrafish and cavefish, and probably in Xenopus too. It could well be the case in mice too.

6. I do not think the GO term data adds much to Figure 1. If possible, I would move it to the supplementary section.

7. It should be made clear which set of experiments was performed as biological replicates and which did not.

8. Based on the heatmap in Fig1A, expression of Rax is significant in GFP- cells at days 4 and 5. The authors should comment or discuss this.

9. Ln 99 of materials and methods mentions that the sorting of GFP+ was performed "when possible". The authors should state the differences in the conditions in the different experiments.

10. The sentence closing the first section of the results (Ln 270) is an overstatement and should be moderated. I cannot see how the results shown in this section on their own could reflect and drive solid conclusions on brain cell fate specification.

11. Appropriate citations should be added to back up the argument that opens the second part of the results section (starting Ln 279).

12. Ln 342-343. I suggest being consistent and using the EF-up or EF-down nomenclature on the

© 2023. Published by The Company of Biologists under the terms of the Creative Commons Attribution License (https://creativecommons.org/licenses/by/4.0/).

whole manuscript unless referring to a different subset of genes.

13. Ln 692 Refers to Fig.S4F, but this figure has only panels A-D.

14. Figures 6B and E and the figure legend do not indicate the differences between the panels, or the time stage of the experiments.

Referees cross-commenting

I agree with the comments and suggestions made by the other two reviewers, which identify similar and also specific issues in the manuscript. I believe they are all pertinent and should be acknowledged before re-submitting.

Significance

The manuscript by Owen et al, presents the analysis of in vitro eye vesicle organoids derived from mouse ESCs at stages equivalent to when the eye field is specified in vivo. This work is pertinent and necessary as detailed data on gene expression in early eye organoids was missing in the field and is necessary for the interpretation of experiments in these systems.

Although the computational data provided in this manuscript is based on consensus TF motifs, the functional relevance of the specific motifs must be proven before being able to drive any significant conclusions, and one should be moderate about the conclusion that can be driven from this kind of analysis. Still, the analysis put forward is a good reference and starting point for future functional studies. One possible limitation of this study is that the quantification of the expression of genes is based on the RNAseq data, and the expression data should be further confirmed using a proper quantitative method like qPCR.

This study will be of interest to the audience studying eye development and disease in animal model systems and humans.

My lab studies the genetic, cellular, and molecular aspects of eye specification, development and disease in zebrafish, and study mutations identified patients with eye globe defects.

Reviewer 3

Evidence, reproducibility and clarity

Studies in Xenopus embryos have established that the specification of the eye field requires a core set of transcription factors (TFs) that impose eye identity to anterior neural plate progenitors. In this manuscript the authors have used mouse embryonic stem cells-derived optic vesicle organoid to ask if the acquisition of mammalian eye identity requires the same set of TFs. They further use different genomic approaches to identify the cis-regulatory elements involved in the expression of these genes and analyses the consequences of altering the sequence of some of the identified regulatory elements. Their results confirm that in mammals the acquisition of eye field identity requires the upregulation of the expression of the same core set of TFs described in Xenopus, with a particularly important role for three of them: Rax, Pax6 and Lhx2. This upregulation is associated to the downregulation of pluripotency genes.

This is a generally well-performed study, that indeed involves a large amount work and adds the identification of several cis-regulatory elements controlling the expression of this core set of already identified eye field TFs. However, conceptually the study does not add much to what is already known and the authors do not offer any very original conclusion from their study. They have generated a large amount of information that likely could allow them to go beyond what is known. For example, they could enlarge the composition of the gene regulatory network that controls eye field specification, given than one of their argument is that their analysis can predict the composition of such a network. Perhaps, they could also address some of the questions that are posed in the discussion. This will strengthen the manuscript and valorize their work.

© 2023. Published by The Company of Biologists under the terms of the Creative Commons Attribution License (https://creativecommons.org/licenses/by/4.0/).

Additional points that could be taken into consideration are the following:

1. According to the text, the authors identify only 53 CREs with decreased chromatin accessibility (ATACseq signal) between the 3 day and 5 days timepoints, versus the 7752 CREs with increased signal. However, this contrasts with the proportion of genes upregulated/ downregulated in their RNAseq analysis (37 vs 448) and with the notion that specification of the eye field involves the concomitant repression of other neural fates. This also suggests that at least an important fraction of the dynamic ATACseq peaks associated with 161 of the 448 downregulated genes increase their accessibility and allow the recruitment of transcriptional repressors. However, the role of TF binding and chromatin accessibility dynamics on gene repression is poorly discussed and the authors need to provide some interpretation of these observations. Also, authors interpret the fact that the presence of BS for EF downregulated genes, such as En2 and GATA6, correlates with increased chromatin accessibility as a consequence of the fact that TFBS can be bound by different TF paralogs but do not seem to consider that these TFs have been reported to work as transcriptional repressors, so that their downregulation could well explain the changes in chromatin accessibility.

2. ATACseq signal analysis is an indirect measure of TF binding. The authors demonstrate the predictive nature of this analysis of TF dynamics and have use an available Sox2 ChIP dataset. However, this does not allow assessing dynamic changes in the occupancy of this TF and its correlation with ATACseq. Therefore, at least for few of the TF stressed in this work (e.g. Sox2 and Otx2 and for which good antibodies exist) they could attempt ChIP-seq analysis. This would considerably strenghen the work and provide support to an idea that the authors have particularly emphasized in their manuscript.

3. Previous studies (i.e. 10.1242/dev.067660; 10.1093/hmg/ddt562) have shown the importance of gene dosage in eye field specification and repression of other fates. These studies could be included in the discussion, which, in its current version is a quite brief and leaves out many of the reported analysis.

Referees cross-commenting

The comments from the other reviewers complement the aspects that we have underscored and should be fully considered as they will contribute to improve the manuscript

Significance

This is a generally well-performed study, that indeed involves a large amount work and adds the identification of several cis-regulatory elements controlling the expression of this core set of already identified eye field TFs. However, conceptually the study does not add much to what is already known and the authors do not offer any very original conclusion from their study. They have generated a large amount of information that likely could allow them to go beyond what is known.

Developmental neurobiologists, genome

Author response to reviewers' comments

[The "revision plan" should delineate the revisions that authors intend to carry out in response to the points raised by the referees. It also provides the authors with the opportunity to explain their view of the paper and of the referee reports.

The document is important for the editors of affiliate journals when they make a first decision on the transferred manuscript. It will also be useful to readers of the reprint and help them to

© 2023. Published by The Company of Biologists under the terms of the Creative Commons Attribution License (https://creativecommons.org/licenses/by/4.0/).

obtain a balanced view of the paper.

If you wish to submit a full revision, please use our "[Full Revision]" template. ***It is important to use the appropriate template to clearly inform the editors of your intentions.]***

1. General Statements [optional]

This section is optional. Insert here any general statements you wish to make about the goal of the study or about the reviews.

We thank all three reviewers for their thoughtful and rigorous critique of our manuscript, which we feel has significantly improved the presentation of our work. In the sections below we (a) outline plans for further analysis to address a few of the more major reviewer comments (where this is possible), (b) detail point-by-point changes we have already made addressing the majority of minor and some major comments by the three reviewers, and (c) point out that we are unfortunately unable to perform any further laboratory work requested (research group closure due to retirement of Prof. FitzPatrick).

Specification of the eye-field during gastrulation represents the earliest known stage of eye development. Using an optic-vesicle organoid model system, the overall goal of our work is to provide an unbiased characterisation of this critical, early developmental event in mammals and to gain insights into relevant gene regulatory mechanisms. A common theme to some of the reviewer comments is that this work doesn't provide much of an advance to the field and our findings are not particularly original. We feel that these comments are slightly harsh for the following reasons. Firstly, although some of our findings are not unexpected, to our knowledge, this is the first unbiased characterisation of the eye-field in a mammalian model system, and not based on knowledge gained through previous work in other non-mammalian vertebrate systems, e.g. Xenopus. Secondly, by generating both RNA-seq and ATAC-seq from a timecourse of organoid development we have been able to quantify dynamic patterns of gene- expression as the eye-field is established and simultaneously gain insights to the regulatory role of some of the key transcription factors, both of which are not present in the literature. Thirdly, by constructing careful, integrated analyses of our RNA-seq and ATAC-seq datasets we were able to generate specific hypotheses regarding cis- regulation of key genes, which we have then demonstrated are possible to efficiently test within the organoid system. In all, although we have been purposely careful not to overinterpret our results, we feel our work does represent a significant step towards understanding the *mammalian* eye field and additionally provides important datasets as well as an analysis framework to begin to quantitatively probe the regulatory mechanisms underlying the transition to an ocular fate. Given the relevance of this developmental event to clinical genetics research as well as to developmental biology we are confident that this work represents an important and significant advance to the literature.

2. Description of the planned revisions

Insert here a point-by-point reply that explains what revisions, additional experimentations and analyses are planned to address the points raised by the referees.

In the following section we outline plans to address three of the more major comments made by the reviewers.

- ***De novo* motif discovery.** 2 out 3 reviewers have pointed essentially to the fact that the presence of a TF motif is not necessarily indicative of TF binding. We are of course aware of this and have therefore restricted our motif analysis to motifs of TFs that are expressed in the organoid timecourse, in addition to carrying out comprehensive footprinting analyses, and have been purposely careful with our working not to overstate the conclusions we make. While it is impossible for us to carry out more wet-lab work, we plan to additionally carry out *de novo* motif discovery and compare our findings to available databases. These analyses will serve as an orthogonal sanity check on whether using the TF-motif databases for the analysis of our biological questions is robust.

© 2023. Published by The Company of Biologists under the terms of the Creative Commons Attribution License (https://creativecommons.org/licenses/by/4.0/).

- **TCF motif analysis.** Reviewer #2 has pointed to the important fact that Tcf's are crucial regulators of Wnt signalling during forebrain specification, and as such deserve to be analysed in the same manner as we did for Sox2 and Otx2. We plan to further analyse our data to check for enrichments in Tcf motifs and evidence of differential Tcf-binding across the organoid timecourse, and report the findings in a revised manuscript.

- **Exploration of further publicly-available ChIP-seq data.** Reviewer #3 has suggested performing ChIP-seq experiments to validate some of our findings and possibly add further evidence to some of the hypotheses we make. We are in the unfortunate position where no further wet-lab work can be performed, so we will not be able to carry out this recommendation. However, we plan to more extensively explore whether there are ChIP-seq datasets, including for Otx2 and Sox2, relevant to early eye/retinal biology that can be analysed to further support the findings and our ideas in the current manuscript.

3. Description of the revisions that have already been incorporated in the transferred manuscript

Please insert a point-by-point reply describing the revisions that were already carried out and included in the transferred manuscript. If no revisions have been carried out yet, please leave this section empty.

In the following section we have copied the comments from each reviewer (in black text) and indicated where we have made changes to the updated manuscript to address these (in green text). We have attempted to address some of the major comments and the majority of minor comments in the updated text. Our plans to address the additional major comments (where this work is possible for us) have been outlined above in Section 2.

Reviewer #1 (Evidence, reproducibility and clarity (Required)):

Summary

Owen et al. characterize the transcriptome and chromatin accessibility of mouse retinal organoids at early stages during which eye field-like cells are specified. Since cell specification and differentiation in retinal organoids largely mimic those processes in vivo, retinal organoids are viable models for studying the mechanisms of early eye development. Owen et al. utilize a previously established Rx-GFP cell line, bulk RNA sequencing, and bulk ATAC sequencing to dissect the mechanisms of early eye development in mice. Their findings are generally consistent with previous studies. Overall, the study is interesting for the field, but its conceptual and technical advances are moderate. In addition, a few major points need to be clarified.

Major points

1. The authors did not show any analysis of retinal organoids at stages when Vsx2 is expressed. This is a significant weakness since the chemically defined medium (CDM) used in Owen et al.'s study was previously shown to induce rostral hypothalamic differentiation (Wataya et al., 2008). Related to this notion, several eye-field transcription factors, such as Rax and Six3, are also expressed in the hypothalamus. Therefore, Owen et al. need to demonstrate that organoids in their modified differentiation system efficiently produce Vsx2-positive retinal progenitors, and samples of organoids at stages when Vsx2 is expressed should be included for RNA sequencing. If Vsx2 is not efficiently expressed in their organoids, the interpretation of results will be very different.

We thank the reviewer for their important comments here. There are several reasons why we are confident that our data and conclusions regarding the organoid eye-field are robust. Firstly, our RNA-seq data, in particular the differences between GFP-positive and GFP-negative cells, clearly show a coordinated up-regulation of the *set* of canonical eye-field TFs (not individually), which previous studies in Xenopus have shown is a prerequisite for differentiation into anterior eye

© 2023. Published by The Company of Biologists under the terms of the Creative Commons Attribution License (https://creativecommons.org/licenses/by/4.0/).

structures (including retina). Secondly, we have checked that some of the later (in development) eye markers, *including Vsx2*, are differentially up-regulated (DeSeq2, logfc>1.5, FDR<10^-3) between the GFP positive and negative cells on day5 (we have added two sentences discussing this and have included a plot of several of these markers in Fig.S2C). This indicates that already on day5 of the timecourse, the RaxGFP-expressing cells are also expressing genes marking later eye development. Interestingly, we have also further analysed our data from the CRISPR-perturbed organoids and found that *Vsx2* (as well as *Vax2*) is also significantly lower expressed in mutant versus wildtype organoids (we have inserted a comment on this in the manuscript text).

In all, we are very confident that our approach of using the optic-vesicle organoids and generating molecular data from an organoid developmental timecourse (including sorting), is unpicking the ocular-fate transition event that we are interested in.

2. The authors state that "two differentiation medias were used for this work due to the differentiation becoming unstable after the initial experiments had been performed. The organoids used for RNA and ATAC-seq were grown in CDM media and the organoids with mutations introduced in potential CREs were grown in KSR media". Why the differentiation becomes unstable after the initial experiments? Differences in the two media cause additional complexities. Related to this notion, "WT Rx-GFP" in Figure 4B and 4E appears to show a different expression pattern compared to that in Figure 1A.

We were unable to identify the reason behind the destabilisation of differentiation in CDM media after the cell lines had been through CRISPR despite thorough testing. The differentiation of these cell lines was stabilised enough using KSR media such that every batch of organoids grown contained some organoids that expressed GFP in a pattern similar to what we had seen before and we carried on our experiments using this. We recognise that using two different media adds complexity, however we see the same patterns of organoid growth and GFP expression when differentiating untransfected WT Rax-GFP cells in both of these medias. We have edited Fig.S1 to include representative images of organoids grown in KSR media which can be directly compared to those grown in CDM shown in Figure 1A.

The reviewer has pointed out that the WT Rx-GFP organoids in Figure 6B and 6E show a different expression pattern to those in figure 1A. With the addition of the supplemental figure mentioned above it becomes apparent that these differences are not due to the change of media. We have clarified in the text that these WT cells have also been transfected so as to act as appropriate controls that have been treated identically to the CRISPR edited cell lines and that this has affected their differentiation capacity.

3. Is the deletion of Rax and Six6 regulatory elements homozygous? Sanger sequencing or amplicon sequencing is needed to show the deletion.

The deletions are homozygous (we have stated this in the manuscript text) and as suggested we have added a supplementary figure showing the Sanger sequencing traces for the WT and mutant cell lines used in this study.

4. The deletion of Rax and Six6 regulatory elements appears to cause minor changes in the expression of Rax and Six6 (Figure 6C, F). Therefore, the impact of findings in bulk RNA seq and bulk ATAC seq in this study is still unclear.

We have added a sentence to the text underlining that developmental genes are expected to be regulated by *multiple* enhancers. Our expectation is therefore, that in perturbing a single putative regulatory element for *Rax/Six6*, we will very likely not see the complete ablation of *Rax/Six6* expression.

5. Retinal organoids and sorted cells are composed of heterogeneous cell populations. Bulk RNA seq and bulk ATAC seq do not have the power to dissect the complexity of heterogeneous cell populations. Single-cell RNA seq and single-cell ATAC seq are more powerful for this study.

© 2023. Published by The Company of Biologists under the terms of the Creative Commons Attribution License (https://creativecommons.org/licenses/by/4.0/).

We agree with the referee about the fact that the organoids are likely composed of relatively heterogeneous cell populations. We have added this limitation of our generated datasets in a “limitations” paragraph in the discussion.

6. Numerous motifs in the JASPAR database are identified using in vitro assays and have not been validated using in vivo assays. Unexpected results in motif analysis could be due to the differences in DNA binding motifs between in vitro and in vivo conditions. This notion should be added in the discussion.

We have added a couple of sentences in the discussion section, highlighting that TF- motif and footprinting analyses of ATAC-seq data provide indirect evidence of TF binding, and to validate these findings experiments such as ChIP-seq or Cut&Run could be performed in the future.

Minor points

Numerous labels in figures are too small.

We have adjusted the size of a number of the figures to increase the size of the labels, which are now mostly the same size as the text in the corresponding figure captions. We are very happy to make further increases in the sizes of figure labels/text upon recommendation.

CROSS-CONSULTATION COMMENTS

My fellow reviewers identify similar major weaknesses and additional points. I agree with the other reviewers' comments.

Reviewer #1 (Significance (Required)):

Nature and Significance of the advances

In Owen et al.'s study, the Rx-GFP cell line and retinal differentiation protocol were established in previous studies (Wataya et al., 2008; Eiraku et al., 2011); bulk RNA sequencing and bulk ATAC sequencing are standard procedures. Although candidate regulatory elements for early eye development are identified, deletions of two prioritized elements using CRISPR/Cas9 only cause minor changes in the expression of targeted genes. Overall, conceptual and technical advances in Owen et al.'s study are moderate.

Compare to existing published knowledge

The datasets could be useful for the field, but conceptual and technical advances are moderate.

Reviewer #2 (Evidence, reproducibility and clarity (Required)):

The authors grow eye organoids from cells with a reporter driving GFP in the Rax locus, a gene that is expressed in the eye field in many animal model systems. They show that expression of GFP picks up by day 4 and performed FACS sorting of GFP+ cells on day 4 and day 5 organoids to compare gene expression by RNAseq comparing with earlier day organoids. The data shows 37 genes with a differential expression on days 4 and 5, compared to day 3, and enriched in GFP+ cells, which they define as EF-up genes. It is notable that some of these genes had already been identified as canonical eye field gene regulatory network transcription factors. In the same way, they identify a group of differentially expressed regulated genes, EF-down, and state that 'many' of them are involved in pluripotency. However, they do not mention how many, or the proportion of these genes in the whole list.

The number of EF-down genes with GO terms linked to pluripotency has now been added to the text.

It would be useful if they could provide the number to understand how many of these genes are related to pluripotency, the whole list of genes mentioned to be downregulated in a

© 2023. Published by The Company of Biologists under the terms of the Creative Commons Attribution License (https://creativecommons.org/licenses/by/4.0/).

supplementary file.

We appreciate that this list was missing and will include it now as a supplemental file.

The authors also note that genes known to be required for eye specification like Sox2 and Otx2 are not differentially expressed across the day 3-4 timepoint (Ln 190).

However, this is not surprising considering that both genes are broadly expressed in the anterior neural ectoderm and required for its specification, which should be noted by the authors.

We have amended the aforementioned sentence to reflect this:

"It is noteworthy that *Sox2* and *Otx2*, known to be crucial in eye development are not differentially expressed across this critical time-point (Fig.2A), consistent with these genes being more broadly expressed in the anterior neuroectoderm *in vivo*."

The authors then go on and cluster the EF-up, EF-down and genes deferentially expressed between days 2 and 3, and identify 6 discreet trajectory groups. From this analysis, they identify a third group of genes which shows a peak on day 3 but whose expression falls on days 4 and 5. It is interesting to see that this group includes Wnt and Fgf morphogenes. The authors should provide a list of the genes in the different clusters for the readers to inspect and analyse.

This information is now available in a supplementary file.

Aiming to generate insight into the cis-regulatory elements that regulate of the genes the authors found differentially expressed in their model system they performed a series of ATAC-seq experiments. When linking the genomic regions with differential ATAC-seq accessibility to gene locus using the GREAT analysis, they identified association to 22 of the EF-up and 161 of the EF-down genes. This suggests a functional link between the ATAC-seq genomic regions and the gene regulation of the differentially expressed genes.

The authors later screened the ATAC-seq regions of increased accessibility for TF binding motifs and found that these regions were enriched with motifs for EFTF genes Rax, Lhx2 and Pax6. When assessing motifs in the ATAC-seq regions in EF-up TADs, Rax and Lhx2 motifs scored highly associated to open chromatin positions. Authors also observe a positive gene expression-accessibility correlation between in Pax6, Lhx2, Six3 and Otx2, and suggest this could mean these genes activate transcription of the EF-up group of genes. The same analysis, but focusing on EF-down genes, suggests that EFTFs repress the expression of EF-down genes which include those involved in pluripotency.

Further interrogating the ATAC-seq data, the authors use TOBIAS footprinting analysis to identify changes in TF binding in EF-TADs and EF-up motifs. Remarkably, whole genome analysis reveals that the largest increase in motif binding corresponds to EF-up genes Rax, Pax6 and Lhx2. The authors then narrow down on specific gene regulation by studying the ATAC-seq data within the TAD of Rax and Six6. However, they do not explain the rationale for which these two genes were highlighted, and why Pax6 or Lhx2 were excluded. This explanation should be added to the manuscript.

We have expanded this section of the manuscript to explain that Rax and Six6 were prioritised due to the GFP readout of Rax expression and Six6 being located in a smaller and thus less complex TAD than Pax6, Six3 and Lhx2 after the initial analysis was performed for all five TADs.

The analysis identifies three regulatory elements in the Rax TAD and two for Six6. They then go on and study one putative regulatory element of each gene and generate CRISPR deletions in cell lines. The rationale for the choice of these particular elements is not clear, nor if the cell lines are the same used for the RNAseq experiments. This information should be explicit in the results and in the methods section.

The manuscript has been updated to include the rationale behind our choice of the regulatory elements deleted.

© 2023. Published by The Company of Biologists under the terms of the Creative Commons Attribution License (https://creativecommons.org/licenses/by/4.0/).

The authors mention that the CRISPR cell lines are "considerably more variable" (Ln 822) compared to the previously studied organoids and suggest that no conclusions can be driven from GFP expression or morphology alone. However, they do not specify which is the variable trait. This information should be added to the text.

We have amended the text to include that the organoids are more variable in terms of the OV like structures produced and GFP expression level.

The authors also miss out on specifying the time stage of the organoids in figure 6 which should be stated.

We thank the reviewer for pointing this out and have updated the manuscript to contain the stage of the organoids.

Regardless, the wildtype organoids in figure 6 and figure S7 show a very different morphology and GFP expression compared to those in figure 1, suggesting that the conclusions from this last set of experiments are not reliable or comparable to those in figure 1. This, together with the fact that different reagents were used to grow the organoids for the RNAseq and the CRISPR experiments, is a weakness of this work that must be addressed.

We recognise this weakness however our amendments detailed above in response to reviewer 1's comments, including adding a figure showing WT organoids grown in the KSR media that closely resemble the organoids in Fig.1A, removes the uncertainty that it is the change in media producing these differences in morphology and GFP expression.

Our aim in this section was to specifically test the hypotheses regarding the regulatory nature of the distal genomic regions identified by our intra-TAD analyses of ATAC-seq data. To do this it was important to compare organoids derived from wildtype and mutant cells that had been subjected to the same growth conditions and genomic-editing protocols. The stress associated with the latter is what we expect has resulted in the differences in morphology and GFP expression compared with the original Fig1. organoids (which have not been through this procedure).

The last part of the results section belongs to the discussion as no results generated by the researchers are included.

Although no new data was generated for this section, we have used the data generated in our work, together with existing ChIP-seq datasets to construct a new plausible hypothesis regarding the activation of Rax-expression through changes in TF-binding at an enhancer displaying little/no change in accessibility. As this section ties in with previous results sections discussing the regulation of eye-field genes, we feel it belongs in the results section rather than in the discussion.

The discussion in this paper is a good opportunity to state the limitation of this study.

As requested, we have added a paragraph discussing the main limitations to our study in the discussion section.

Major comments to address

1. One of the main issues identified is that the morphology of the control conditions in the CRISPR experiments (Fig.6) do not look is that those used for the RNAseq experiments (Fig.1) and the authors should address this issue. The fact that CDM media was used on the RNA extraction and ATACseq experiments and then KSR media was used for the CRISPR experiments is worrying and makes one wonder whether the second set of experiments is at all comparable to the first. This should be somehow controlled carefully by at least replicating one set of RNA experiments with the KSR media.

We have addressed this in response to the reviewer's summary above. Unfortunately, it is not

© 2023. Published by The Company of Biologists under the terms of the Creative Commons Attribution License (https://creativecommons.org/licenses/by/4.0/).

possible for us to replicate the RNA experiments in the KSR media due to the research group closure upon Professor FitzPatrick's retirement.

2. The requirement of Wnt signalling inhibition has been well established as a requirement for forebrain specification, including the eye field. Considering the link of the Wnt/beta-catenin pathway to eye specification and that TCFs, the transcription factors that mediate Wnt pathway transcription regulation, have known and well-studied DNA motifs, it is surprising that authors do not include the analysis of TCF motifs in their study. Also considering that TCF7l1 (TCF3, old nomenclature) has recently been shown to be cell-autonomously required for the expression of rx3 (Rax homologue) in zebrafish. One would expect TCFs to be included in the analysis as it was done with Sox2 and Otx2, which were studied due to the known relevance in forebrain specification rather than from the direct analysis of the differential gene expression experiments.

We thank the referee for their valuable comment here. Our current analyses indeed do not consider TCFs and are therefore likely incomplete. We plan to address this (section 2 of Revision Plan) by further analysing our data to quantify the patterns and effects of the TCF genes, and will appropriately amend our manuscript to reflect our findings.

Minor comments to address

1. The authors should clearly state the day timepoint used in the organoids experiments in the results section and figure legends, not just in the methods.

We have updated the text and figure legends to include the time point of all organoids.

2. The report by Agnes et al Development 2022 should be cited in the introduction as it is an excellent paper related to this topic, including a comprehensive analysis of the EFTFs expression pattern.

We thank the reviewer for pointing us to this very interesting paper. Although we feel it doesn't fit in with our introduction that is currently tailored to the set of genes that has historically defined the eye-field (and which was discovered in non-mammalian models), we do recognise that the 3D organisation of the eye-field and in particular the patterns of gene-expression defining different regions of this is important to disentangle in mammalian systems. We have therefore inserted a reference to the Agnes at al 2022 study on the dimorphic teleost in our extended discussion.

3. Ln 41. Mutations in these genes do not always cause severe bilateral eye malformations. Probably best to moderate and mention that they 'can' cause these malformations.

As suggested we have softened this sentence to:

"Mutations in at least three of the genes encoding orthologs of the Xenopus EFTF ***can*** cause severe bilateral eye malformations in humans (OTX2, PAX6 and RAX) (Fitzpatrick and van Heyningen, 2005)."

4. Ln 146. Authors mention that in vitro organoid systems "closely mimic the in vitro regulatory dynamics". This statement should be moderated as we do not know if this is true. In fact, one of the positive aspects of this study is that it contributes to supporting this statement.

We agree with the referee regarding the strength of this original statement. We have changed this to:

"We have exploited a reproducible, *in vitro* organoid model system enabling us to generate data from this cell-state transition and through computational analysis gain a quantitative understanding of the underlying regulatory mechanisms."

5. Ln 150. Rax homologue Rx3 is also expressed in cells that give rise to the hypothalamus in zebrafish and cavefish, and probably in Xenopus too. It could well be the case in mice too.

© 2023. Published by The Company of Biologists under the terms of the Creative Commons Attribution License (https://creativecommons.org/licenses/by/4.0/).

We have corrected this to indicate that Rax is also expressed in the hypothalamus in mice.

6. I do not think the GO term data adds much to Figure 1. If possible, I would move it to the supplementary section.

We have moved the GO visualisations to supplementary, Fig.S2.

7. It should be made clear which set of experiments was performed as biological replicates and which did not.

We have added details on the number of replicates used in each experiment.

8. Based on the heatmap in Fig1A, expression of Rax is significant in GFP- cells at days 4 and 5. The authors should comment or discuss this.

We have amended the text and supplemental methods section to include more details of our FACS protocol. The limitations of our sorting procedure include the fact that cells are not sorted into pure GFP expressing and non-expressing populations. Rather the GFP negative sample may contain some cells with low Rax expression or cells that have just begun to express Rax that were not excluded by our sorting. Our aim was to collect sufficient numbers of cells for each condition and separate out cells that expressed GFP to get a more uniform population of cells to study.

It is also of note that the heatmap shows Rax expression by day 3. Although it was not detectable by imaging there were around 100 cells per organoid that FACS marked as GFP positive but were retained within the day 3 sample to ensure we had a complete picture of the gene expression at this time point.

9. Ln 99 of materials and methods mentions that the sorting of GFP+ was performed "when possible". The authors should state the differences in the conditions in the different experiments.

This has been expanded to detail exactly how cells were sorted.

10. The sentence closing the first section of the results (Ln 270) is an overstatement and should be moderated. I cannot see how the results shown in this section on their own could reflect and drive solid conclusions on brain cell fate specification.

We agree with the referee and have changed this sentence to:

"In summary, these first analyses of RNA-seq data generated from the timecourse of optic vesicle organoid development, show that this is a robust and relevant model system with which to study the gene dynamics underlying mammalian eye field specification."

11. Appropriate citations should be added to back up the argument that opens the second part of the results section (starting Ln 279).

We have added several citations that discuss and review the current knowledge regarding gene regulation via TF-binding at accessible cis-regulatory elements.

12. Ln 342-343. I suggest being consistent and using the EF-up or EF-down nomenclature on the whole manuscript unless referring to a different subset of genes.

We have modified the text to consistently use "EF-up" or "EF-down" terminology.

13. Ln 692 Refers to Fig.S4F, but this figure has only panels A-D.

This was a typo and has been corrected in the text.

14. Figures 6B and E and the figure legend do not indicate the differences between the panels, or the time stage of the experiments.

© 2023. Published by The Company of Biologists under the terms of the Creative Commons Attribution License (https://creativecommons.org/licenses/by/4.0/).

The figure legend has been updated to include these details.

CROSS-CONSULTATION COMMENTS

I agree with the comments and suggestions made by the other two reviewers, which identify similar and also specific issues in the manuscript. I believe they are all pertinent and should be acknowledged before re-submitting.

Reviewer #2 (Significance (Required)):

The manuscript by Owen et al, presents the analysis of in vitro eye vesicle organoids derived from mouse ESCs at stages equivalent to when the eye field is specified in vivo. This work is pertinent and necessary as detailed data on gene expression in early eye organoids was missing in the field and is necessary for the interpretation of experiments in these systems.

Although the computational data provided in this manuscript is based on consensus TF motifs, the functional relevance of the specific motifs must be proven before being able to drive any significant conclusions, and one should be moderate about the conclusion that can be driven from this kind of analysis. Still, the analysis put forward is a good reference and starting point for future functional studies. One possible limitation of this study is that the quantification of the expression of genes is based on the RNAseq data, and the expression data should be further confirmed using a proper quantitative method like qPCR.

This study will be of interest to the audience studying eye development and disease in animal model systems and humans.

My lab studies the genetic, cellular, and molecular aspects of eye specification, development and disease in zebrafish, and study mutations identified patients with eye globe defects.

Reviewer #3 (Evidence, reproducibility and clarity (Required)):

Studies in Xenopus embryos have established that the specification of the eye field requires a core set of transcription factors (TFs) that impose eye identity to anterior neural plate progenitors. In this manuscript the authors have used mouse embryonic stem cells-derived optic vesicle organoid to ask if the acquisition of mammalian eye identity requires the same set of TFs. They further use different genomic approaches to identify the cis-regulatory elements involved in the expression of these genes and analyses the consequences of altering the sequence of some of the identified regulatory elements. Their results confirm that in mammals the acquisition of eye field identity requires the upregulation of the expression of the same core set of TFs described in Xenopus, with a particularly important role for three of them: Rax, Pax6 and Lhx2. This upregulation is associated to the downregulation of pluripotency genes.

This is a generally well-performed study, that indeed involves a large amount work and adds the identification of several cis-regulatory elements controlling the expression of this core set of already identified eye field TFs. However, conceptually the study does not add much to what is already known and the authors do not offer any very original conclusion from their study. They have generated a large amount of information that likely could allow them to go beyond what is known. For example, they could enlarge the composition of the gene regulatory network that controls eye field specification, given than one of their argument is that their analysis can predict the composition of such a network. Perhaps, they could also address some of the questions that are posed in the discussion. This will strengthen the manuscript and valorize their work.

Additional points that could be taken into consideration are the following:

1) According to the text, the authors identify only 53 CREs with decreased chromatin accessibility (ATACseq signal) between the 3 day and 5 days timepoints, versus the 7752 CREs with increased signal. However, this contrasts with the proportion of genes upregulated/ downregulated in their RNAseq analysis (37 vs 448) and with the notion that specification of

© 2023. Published by The Company of Biologists under the terms of the Creative Commons Attribution License (https://creativecommons.org/licenses/by/4.0/).

the eye field involves the concomitant repression of other neural fates. This also suggests that at least an important fraction of the dynamic ATACseq peaks associated with 161 of the 448 downregulated genes increase their accessibility and allow the recruitment of transcriptional repressors. However, the role of TF binding and chromatin accessibility dynamics on gene repression is poorly discussed and the authors need to provide some interpretation of these observations. Also, authors interpret the fact that the presence of BS for EF downregulated genes, such as En2 and GATA6, correlates with increased chromatin accessibility as a consequence of the fact that TFBS can be bound by different TF paralogs but do not seem to consider that these TFs have been reported to work as transcriptional repressors, so that their downregulation could well explain the changes in chromatin accessibility.

We thank the reviewer for their interesting comments here. We have added short discussions on both main points above (EF-down genes linked to peaks with increasing accessibility and En2/Gata as transcriptional repressors) in the text related to the analysis of our ATAC-seq data. The notion that a loss of repression leading to the activation of gene-expression is indeed a very exciting one and one that we have thought about in the context of the switch-on of the eye-field TFs. This certainly deserves further future work, however in the present study we wanted to be careful not to overinterpret our data. To robustly gain insights into the loss of repression, experiments such as En1/Gata6 ChIP-seq would be very useful, though we are unable to perform these in the near future.

2) ATACseq signal analysis is an indirect measure of TF binding. The authors demonstrate the predictive nature of this analysis of TF dynamics and have use an available Sox2 ChIP dataset. However, this does not allow assessing dynamic changes in the occupancy of this TF and its correlation with ATACseq. Therefore, at least for few of the TF stressed in this work (e.g. Sox2 and Otx2 and for which good antibodies exist) they could attempt ChIP-seq analysis. This would considerably strengthen the work and provide support to an idea that the authors have particularly emphasized in their manuscript.

We agree with the referee that not having generated ChIP-seq data does not allow us to validate some of the hypotheses and evidence provided by the computational analysis of our ATAC-seq data – we have added a discussion of this limitation in the discussion section of our manuscript. We do note however, as observed in *Bentsen et al, 2020*, that compared to simple TF-motif occurrence analyses, TF-footprinting analyses (such as those we have performed) yield results on putative TF binding that are much closer to more direct measurements of TF binding via e.g. ChIP-seq.

We fully agree that it would be very interesting to perform ChIP-seq/Cut&Cut experiments on the organoid system for a set of interesting TFs identified in our study. Unfortunately, because the lab of Prof FitzPatrick has now closed, it is not possible for us to perform further wet-lab experiments in the very near future. However, we plan (section 2 of Revision Plan) to further explore the literature to try to find additional publicly-available ChIP-seq datasets (including for Otx2) which would help reinforce some of the hypotheses we make, and will report any relevant findings in our final manuscript.

3) Previous studies (i.e. 10.1242/dev.067660; 10.1093/hmg/ddt562) have shown the importance of gene dosage in eye field specification and repression of other fates. These studies could be included in the discussion, which, in its current version is a quite brief and leaves out many of the reported analysis.

We thank the referee for pointing us to this very relevant question – we have added this to the further research questions in the discussion.

CROSS-CONSULATION COMMENTS

The comments from the other reviewers complement the aspects that we have underscored and should be fully considered as they will contribute to improve the manuscript.

© 2023. Published by The Company of Biologists under the terms of the Creative Commons Attribution License (https://creativecommons.org/licenses/by/4.0/).

Reviewer #3 (Significance (Required)):

This is a generally well-performed study, that indeed involves a large amount work and adds the identification of several cis-regulatory elements controlling the expression of this core set of already identified eye field TFs. However, conceptually the study does not add much to what is already known and the authors do not offer any very original conclusion from their study. They have generated a large amount of information that likely could allow them to go beyond what is known.

Developmental neurobiologists, genome

4. Description of analyses that authors prefer not to carry out

Please include a point-by-point response explaining why some of the requested data or additional analyses might not be necessary or cannot be provided within the scope of a revision. This can be due to time or resource limitations or in case of disagreement about the necessity of such additional data given the scope of the study. Please leave empty if not applicable.

The reviewers have pointed out that some additional laboratory work could be performed to further check and support our findings and the conclusions we present in our manuscript. While we are confident that the experimental work included in this paper is robust (and furthermore we have both been very careful not to overstate our findings as well as provide further evidence and data in our response to address concerns raised by the reviewers), we are unfortunately not in a position to do any further laboratory work. The reason for this is that Prof. FitzPatrick has retired from his position at the MRC HGU and his research group has closed.

Original submission

First decision letter

MS ID#: DEVELOP/2022/201432

MS TITLE: Characterization of an Eye Field-like State during Optic Vesicle Organoid Development

AUTHORS: Liusaidh J Owen, Jacqueline Rainger, Hemant Bengani, Fiona Kilanowski, David R FitzPatrick, and Andrew Sofronis Papanastasiou

Apologies for the delay in coming to a decision about your manuscript, submitted to us through Review Commons. I have now had a chance to read thepaper, the reviews and your response to the reviews and have discussed the manuscript with our executive editor. We both consider that there is a lot of useful novel data in the study that will be of considerable interest and use for the research community. We also feel that the level of conceptual novelty is less than we would expect for a Development paper given related studies in other species. Despite this, we do think the data presented is potentially a very useful resource for future research and so think that the study has more potential as a Techniques and Resources article than a Research Article. We also think that some of the requests for further experimental work from the reviewers to extend the novelty of the study would be less critical for a Resource article. Of course, we cannot be sure how the reviewers will respond after you have completed your revisions but I will explain our reasoning for considering as a Resource article when I ask them to re-review the manuscript.

If you are willing to have your manuscript considered as a Resource Article, please attend to all of the reviewers' comments and ensure that you clearly highlight all changes made in the revised manuscript. Please avoid using 'Tracked changes' in Word files as these are lost in PDF conversion. I should be grateful if you would also provide a point-by-point response detailing how you have dealt with the points raised by the reviewers in the 'Response to Reviewers' box. If you do not agree with any of their criticisms or suggestions please explain clearly why this is so.

© 2023. Published by The Company of Biologists under the terms of the Creative Commons Attribution License (https://creativecommons.org/licenses/by/4.0/).

First revision

Author response to reviewers' comments

Title: Characterization of an Eye Field-like State during Optic Vesicle Organoid Development
Development manuscript ID: DEVELOP/2022/201432
Review Commons Manuscript number: RC-2022-01640
Corresponding author(s): Andrew, Papanastasiou

General Comments

We firstly thank all three reviewers for their thoughtful and rigorous critique of our manuscript, which we feel has significantly improved the presentation of our work. In this document we provide a point-by-point response to each reviewer's comments and suggestions, including full details of the corresponding manuscript amendments and additions we have made.

We appreciate that, in the context of previous work in non-mammalian systems, the main findings of our work are not wholly unexpected. However, we would like to emphasise that, to our knowledge, the study we present is the first unbiased characterisation of the eye-field in a mammalian model system, and not based on knowledge gained through previous work in other non-mammalian vertebrate systems. Although we have been purposely careful not to over-interpret our results, we feel our work does represent a significant step towards understanding the mammalian eye field. It additionally provides important datasets as well as an analysis framework to begin to quantitatively probe the regulatory mechanisms underlying the transition to an ocular fate. Given the relevance of this developmental event to clinical genetics research as well as to developmental biology we are confident that this work represents an interesting and important advance to the literature.

Finally, while we are confident that the experimental work included in this paper is robust, we would like to point out once again that we are unfortunately unable to perform any further laboratory work requested due to closure of the research group (retirement of Prof. FitzPatrick). We feel that we have both been very careful not to overstate our findings and also have made every effort to provide further evidence and data in our response to address concerns raised by the reviewers (that may have been addressed with further experimental work).

Detailed response and description of revisions

In this section we have copied the comments from each reviewer (in black text) and indicated where we have made changes to the updated manuscript to address these (in green text).

Reviewer #1 (Evidence, reproducibility and clarity (Required)):

Summary

Owen et al. characterize the transcriptome and chromatin accessibility of mouse retinal organoids at early stages during which eye field-like cells are specified. Since cell specification and differentiation in retinal organoids largely mimic those processes in vivo, retinal organoids are viable models for studying the mechanisms of early eye development. Owen et al. utilize a previously established Rx-GFP cell line, bulk RNA sequencing, and bulk ATAC sequencing to dissect the mechanisms of early eye development in mice. Their findings are generally consistent with previous studies. Overall, the study is interesting for the field, but its conceptual and technical advances are moderate. In addition, a few major points need to be clarified.

Major points

1. The authors did not show any analysis of retinal organoids at stages when Vsx2 is expressed. This is a significant weakness since the chemically defined medium (CDM) used in Owen et al.'s study was previously shown to induce rostral hypothalamic differentiation (Wataya et al., 2008). Related

© 2023. Published by The Company of Biologists under the terms of the Creative Commons Attribution License (https://creativecommons.org/licenses/by/4.0/).

to this notion, several eye-field transcription factors, such as Rax and Six3, are also expressed in the hypothalamus. Therefore, Owen et al. need to demonstrate that organoids in their modified differentiation system efficiently produce Vsx2-positive retinal progenitors, and samples of organoids at stages when Vsx2 is expressed should be included for RNA sequencing. If Vsx2 is not efficiently expressed in their organoids, the interpretation of results will be very different.

We thank the reviewer for their important comments here. There are several reasons why we are confident that our data and conclusions regarding the organoid eye-field are robust. Firstly, our RNA-seq data, in particular the differences between GFP-positive and GFP-negative cells, clearly show a coordinated up-regulation of the set of canonical eye-field TFs (not individually), which previous studies in Xenopus have shown is a prerequisite for differentiation into anterior eye structures (including retina). Secondly, we have checked that some of the later (in development) eye markers, including Vsx2, are differentially up-regulated (DeSeq2, logfc>1.5, FDR<10^-3) between the GFP positive and negative cells on day5 (we have added two sentences discussing this [line183] and have included a plot of several of these markers in Fig.S2C). This indicates that already on day5 of the timecourse, the RaxGFP-expressing cells are also expressing genes marking later eye development. Interestingly, we have also further analysed our data from the CRISPR-perturbed organoids and found that Vsx2 (as well as Vax2) is also significantly lower expressed in mutant versus wildtype organoids (we have inserted a comment on this in the manuscript text [line937]).

In all, we are very confident that our approach of using the optic-vesicle organoids and generating molecular data from an organoid developmental timecourse (including sorting), is unpicking the ocular-fate transition event that we are interested in.

2. The authors state that "two differentiation medias were used for this work due to the differentiation becoming unstable after the initial experiments had been performed. The organoids used for RNA and ATAC-seq were grown in CDM media and the organoids with mutations introduced in potential CREs were grown in KSR media". Why the differentiation becomes unstable after the initial experiments? Differences in the two media cause additional complexities. Related to this notion, "WT Rx-GFP" in Figure 4B and 4E appears to show a different expression pattern compared to that in Figure 1A.

We were unable to identify the reason behind the destabilisation of differentiation in CDM media after the cell lines had been through CRISPR despite thorough testing. The differentiation of these cell lines was stabilised enough using KSR media such that every batch of organoids grown contained some organoids that expressed GFP in a pattern similar to what we had seen before and we carried on our experiments using this. We recognise that using two different media adds complexity, however we see the same patterns of organoid growth and GFP expression when differentiating untransfected WT Rax-GFP cells in both of these medias. We have edited Fig.S1 to include representative images of organoids grown in KSR media which can be directly compared to those grown in CDM shown in Figure 1A.

The reviewer has pointed out that the WT Rx-GFP organoids in Figure 6B and 6E show a different expression pattern to those in figure 1A. With the addition of the supplemental figure mentioned above it becomes apparent that these differences are not due to the change of media. We have clarified in the main text [line905] that these WT cells have also been transfected so as to act as appropriate controls that have been treated identically to the CRISPR edited cell lines and that this has affected their differentiation capacity.

3. Is the deletion of Rax and Six6 regulatory elements homozygous? Sanger sequencing or amplicon sequencing is needed to show the deletion.

The deletions are homozygous (we have stated this in the manuscript text [lines 896 and 900]) and as suggested we have added a supplementary figure (Fig.S7) showing the Sanger sequencing traces for the WT and mutant cell lines used in this study.

4. The deletion of Rax and Six6 regulatory elements appears to cause minor changes in the expression of Rax and Six6 (Figure 6C, F). Therefore, the impact of findings in bulk RNA seq and bulk ATAC seq in this study is still unclear.

© 2023. Published by The Company of Biologists under the terms of the Creative Commons Attribution License (https://creativecommons.org/licenses/by/4.0/).

We have added a sentence to the text underlining that developmental genes are expected to be regulated by multiple enhancers. Our expectation is therefore, that in perturbing a single putative regulatory element for Rax/Six6, we will very likely not see the complete ablation of Rax/Six6 expression [line923].

5. Retinal organoids and sorted cells are composed of heterogeneous cell populations. Bulk RNA seq and bulk ATAC seq do not have the power to dissect the complexity of heterogeneous cell populations. Single-cell RNA seq and single-cell ATAC seq are more powerful for this study.

We agree with the referee about the fact that the organoids are likely composed of relatively heterogeneous cell populations. We have added this limitation of our generated datasets in a "limitations" paragraph in the discussion [lines1152-1162].

6. Numerous motifs in the JASPAR database are identified using in vitro assays and have not been validated using in vivo assays. Unexpected results in motif analysis could be due to the differences in DNA binding motifs between in vitro and in vivo conditions. This notion should be added in the discussion.

We have added a couple of sentences in the discussion section, highlighting that TF-motif and footprinting analyses of ATAC-seq data provide indirect evidence of TF binding, and to validate these findings experiments such as ChIP-seq or Cut&Run could be performed in the future [lines1163-1171].

Going further, we have performed de-novo motif discovery analyses on the genomic regions displaying large changes across the critical day3-day5 transition and compared these newly-inferred motifs to the JASPAR database (Tables S5, S6 & Methods). We find that the de novo motifs are significantly similar to many of the JASPAR motifs that came out as most enriched in our original motif-enrichment analyses. These new analyses serve as an orthogonal sanity check on whether using the TF-motif databases for the analysis of our biological questions is reasonable [lines405 and 438].

Minor points

Numerous labels in figures are too small.

We have adjusted the size of a number of the figures to increase the size of the labels, which are now mostly the same size as the text in the corresponding figure captions. We are very happy to make further increases in the sizes of figure labels/text upon recommendation.

CROSS-CONSULTATION COMMENTS

My fellow reviewers identify similar major weaknesses and additional points. I agree with the other reviewers' comments.

Reviewer #1 (Significance (Required)):

Nature and Significance of the advances

In Owen et al.'s study, the Rx-GFP cell line and retinal differentiation protocol were established in previous studies (Wataya et al., 2008; Eiraku et al., 2011); bulk RNA sequencing and bulk ATAC sequencing are standard procedures. Although candidate regulatory elements for early eye development are identified, deletions of two prioritized elements using CRISPR/Cas9 only cause minor changes in the expression of targeted genes. Overall, conceptual and technical advances in Owen et al.'s study are moderate.

Compare to existing published knowledge

The datasets could be useful for the field, but conceptual and technical advances are moderate.

© 2023. Published by The Company of Biologists under the terms of the Creative Commons Attribution License (https://creativecommons.org/licenses/by/4.0/).

Reviewer #2 (Evidence, reproducibility and clarity (Required)):

The authors grow eye organoids from cells with a reporter driving GFP in the Rax locus, a gene that is expressed in the eye field in many animal model systems. They show that expression of GFP picks up by day 4 and performed FACS sorting of GFP+ cells on day 4 and day 5 organoids to compare gene expression by RNAseq comparing with earlier day organoids. The data shows 37 genes with a differential expression on days 4 and 5, compared to day 3, and enriched in GFP+ cells, which they define as EF-up genes. It is notable that some of these genes had already been identified as canonical eye field gene regulatory network transcription factors. In the same way, they identify a group of differentially expressed regulated genes, EF-down, and state that 'many' of them are involved in pluripotency. However, they do not mention how many, or the proportion of these genes in the whole list.

The number of EF-down genes with GO terms linked to pluripotency has now been added to the text [line198].

It would be useful if they could provide the number to understand how many of these genes are related to pluripotency, the whole list of genes mentioned to be downregulated in a supplementary file.

We appreciate that this list was missing and will include it now as a supplemental file.

The authors also note that genes known to be required for eye specification like Sox2 and Otx2 are not differentially expressed across the day 3-4 timepoint (Ln 190). However, this is not surprising considering that both genes are broadly expressed in the anterior neural ectoderm and required for its specification, which should be noted by the authors.

We have amended the aforementioned sentence to reflect this:

"It is noteworthy that Sox2 and Otx2, known to be crucial in eye development are not differentially expressed across this critical time-point (Fig.2A), consistent with these genes being more broadly expressed in the anterior neuroectoderm in vivo." [line 205]

The authors then go on and cluster the EF-up, EF-down and genes deferentially expressed between days 2 and 3, and identify 6 discreet trajectory groups. From this analysis, they identify a third group of genes which shows a peak on day 3 but whose expression falls on days 4 and 5. It is interesting to see that this group includes Wnt and Fgf morphogenes. The authors should provide a list of the genes in the different clusters for the readers to inspect and analyse.

This information is now available in a supplementary file.

Aiming to generate insight into the cis-regulatory elements that regulate of the genes the authors found differentially expressed in their model system they performed a series of ATAC-seq experiments. When linking the genomic regions with differential ATAC-seq accessibility to gene locus using the GREAT analysis, they identified association to 22 of the EF-up and 161 of the EF-down genes. This suggests a functional link between the ATAC-seq genomic regions and the gene regulation of the differentially expressed genes.

The authors later screened the ATAC-seq regions of increased accessibility for TF binding motifs and found that these regions were enriched with motifs for EFTF genes Rax, Lhx2 and Pax6. When assessing motifs in the ATAC-seq regions in EF-up TADs, Rax and Lhx2 motifs scored highly associated to open chromatin positions. Authors also observe a positive gene expression-accessibility correlation between in Pax6, Lhx2, Six3 and Otx2, and suggest this could mean these genes activate transcription of the EF-up group of genes. The same analysis, but focusing on EF-down genes, suggests that EFTFs repress the expression of EF-down genes which include those involved in pluripotency.

Further interrogating the ATAC-seq data, the authors use TOBIAS footprinting analysis to identify changes in TF binding in EF-TADs and EF-up motifs. Remarkably, whole genome analysis reveals that

© 2023. Published by The Company of Biologists under the terms of the Creative Commons Attribution License (https://creativecommons.org/licenses/by/4.0/).

the largest increase in motif binding corresponds to EF-up genes Rax, Pax6 and Lhx2. The authors then narrow down on specific gene regulation by studying the ATAC-seq data within the TAD of Rax and Six6. However, they do not explain the rationale for which these two genes were highlighted, and why Pax6 or Lhx2 were excluded. This explanation should be added to the manuscript.

We have expanded this section of the manuscript to explain that Rax and Six6 were prioritised due to the GFP readout of Rax expression and Six6 being located in a smaller and thus less complex TAD than Pax6, Six3 and Lhx2 after the initial analysis was performed for all five TADs [lines837-874].

The analysis identifies three regulatory elements in the Rax TAD and two for Six6. They then go on and study one putative regulatory element of each gene and generate CRISPR deletions in cell lines. The rationale for the choice of these particular elements is not clear, nor if the cell lines are the same used for the RNAseq experiments. This information should be explicit in the results and in the methods section.

The manuscript has been updated to include the rationale behind our choice of the regulatory elements deleted [lines 874-891].

The authors mention that the CRISPR cell lines are "considerably more variable" (Ln 822) compared to the previously studied organoids and suggest that no conclusions can be driven from GFP expression or morphology alone. However, they do not specify which is the variable trait. This information should be added to the text.

We have amended the text to include that the organoids are more variable in terms of the OV like structures produced and GFP expression level [line907].

The authors also miss out on specifying the time stage of the organoids in figure 6 which should be stated.

We thank the reviewer for pointing this out and have updated the manuscript to contain the stage of the organoids.

Regardless, the wildtype organoids in figure 6 and figure S7 show a very different morphology and GFP expression compared to those in figure 1, suggesting that the conclusions from this last set of experiments are not reliable or comparable to those in figure 1. This, together with the fact that different reagents were used to grow the organoids for the RNAseq and the CRISPR experiments, is a weakness of this work that must be addressed.

We recognise this weakness however our amendments detailed above in response to reviewer 1's comments, including adding a figure showing WT organoids grown in the KSR media that closely resemble the organoids in Fig.1A, removes the uncertainty that it is the change in media producing these differences in morphology and GFP expression.

Our aim in this section was to specifically test the hypotheses regarding the regulatory nature of the distal genomic regions identified by our intra-TAD analyses of ATAC-seq data. To do this it was important to compare organoids derived from wildtype and mutant cells that had been subjected to the same growth conditions and genomic-editing protocols. The stress associated with the latter is what we expect has resulted in the differences in morphology and GFP expression compared with the original Fig1. organoids (which have not been through this procedure).

The last part of the results section belongs to the discussion as no results generated by the researchers are included.

Although no new data was generated for this section, we have used the data generated in our work, together with existing ChIP-seq datasets and data from the UniBind TF-DNA interactions database (Puig et al, 2021) to construct a new plausible hypothesis regarding the activation of Rax-expression through changes in TF-binding at an enhancer displaying little/no change in accessibility. As this section ties in with previous results sections discussing the regulation of eye-field genes, we feel it belongs in the results section rather than in the discussion.

© 2023. Published by The Company of Biologists under the terms of the Creative Commons Attribution License (https://creativecommons.org/licenses/by/4.0/).

The discussion in this paper is a good opportunity to state the limitation of this study.

As requested, we have added a paragraph discussing the main limitations to our study in the discussion section [lines 1152-1171].

Major comments to address

1. One of the main issues identified is that the morphology of the control conditions in the CRISPR experiments (Fig.6) do not look is that those used for the RNAseq experiments (Fig.1) and the authors should address this issue. The fact that CDM media was used on the RNA extraction and ATACseq experiments and then KSR media was used for the CRISPR experiments is worrying and makes one wonder whether the second set of experiments is at all comparable to the first. This should be somehow controlled carefully by at least replicating one set of RNA experiments with the KSR media.

We have addressed this in response to the reviewer's summary above. Unfortunately, it is not possible for us to replicate the RNA experiments in the KSR media due to the research group closure upon Prof. FitzPatrick's retirement.

2. The requirement of Wnt signalling inhibition has been well established as a requirement for forebrain specification, including the eye field. Considering the link of the Wnt/beta-catenin pathway to eye specification and that TCFs, the transcription factors that mediate Wnt pathway transcription regulation, have known and well-studied DNA motifs, it is surprising that authors do not include the analysis of TCF motifs in their study. Also considering that TCF7l1 (TCF3, old nomenclature) has recently been shown to be cell-autonomously required for the expression of rx3 (Rax homologue) in zebrafish. One would expect TCFs to be included in the analysis as it was done with Sox2 and Otx2, which were studied due to the known relevance in forebrain specification rather than from the direct analysis of the differential gene expression experiments.

We thank the referee for their valuable comment here. Our previous analyses indeed did not consider TCFs and are therefore likely incomplete. We have addressed this by re-analysing our data now investigating Tcf3, on the same footing as Sox2 and Otx2. We have updated all relevant figures to illustrate the patterns and importance of Tcf3 across the various analyses we have made.

In general, we find that although, like Sox2 and Otx2, Tcf3 does not pass our fold-change and significance thresholds for differential expression, it's binding motifs do show patterns that provide evidence towards it's involvement in the regulation of the eye field, through interactions with distal open-chromatin regions (Fig S2C, Fig 4 and Fig S4E&F).

The de-novo motif discovery analysis we have now performed, reveals an interesting pattern. For the analysis performed on peaks increasing in accessibility, two de novo motifs that are significantly similar to the Tcf3_1 and Tcf3_2 JASPAR motifs (Table S5), whilst for the analysis performed on the peaks with decreasing accessibility, we find a motif similar to the Tcf3 JASPAR motif (Table S6). This observation is not one we have been able to explain fully but may suggest that Tcf3 interacts with different co-binding TFs at these loci, which result in it being involved in eye field specification through a combination of diverging effects (activating/suppressing). We have not been able to disentangle these effects in further detail so have purposefully not overstated any conclusions regarding this in the text. This does indicate an interesting future direction of work regarding investigating the relationship between the diverging activity of these loci and the diverging binding of Tcf3.

Minor comments to address

1. The authors should clearly state the day timepoint used in the organoids experiments in the results section and figure legends, not just in the methods.

We have updated the text and figure legends to include the time point of all organoids.

© 2023. Published by The Company of Biologists under the terms of the Creative Commons Attribution License (https://creativecommons.org/licenses/by/4.0/).

2. The report by Agnes et al Development 2022 should be cited in the introduction as it is an excellent paper related to this topic, including a comprehensive analysis of the EFTFs expression pattern.

We thank the reviewer for pointing us to this very interesting paper. Although we feel it doesn't fit in with our introduction that is currently tailored to the set of genes that has historically defined the eye field (and which was discovered in non-mammalian models), we do recognise that the 3D organisation of the eye field and in particular the patterns of gene expression defining different regions of this is important to disentangle in mammalian systems. We have therefore inserted a reference to the Agnes at al 2022 study on the dimorphic teleost in our extended discussion [line 1181].

3. Ln 41. Mutations in these genes do not always cause severe bilateral eye malformations. Probably best to moderate and mention that they 'can' cause these malformations.

As suggested we have softened this sentence to:

"Mutations in at least three of the genes encoding orthologs of the Xenopus EFTF can cause severe bilateral eye malformations in humans (OTX2, PAX6 and RAX) (Fitzpatrick and van Heyningen, 2005)." [line41]

4. Ln 146. Authors mention that in vitro organoid systems "closely mimic the in vitro regulatory dynamics". This statement should be moderated as we do not know if this is true. In fact, one of the positive aspects of this study is that it contributes to supporting this statement.

We agree with the referee regarding the strength of this original statement. We have changed this to:

"We have exploited a reproducible, in vitro organoid model system enabling us to generate data from this cell-state transition and through computational analysis gain a quantitative understanding of the underlying regulatory mechanisms." [line112]

5. Ln 150. Rax homologue Rx3 is also expressed in cells that give rise to the hypothalamus in zebrafish and cavefish, and probably in Xenopus too. It could well be the case in mice too.

We have corrected this to indicate that Rax is also expressed in the hypothalamus in mice. [line151]

6. I do not think the GO term data adds much to Figure 1. If possible, I would move it to the supplementary section.

We have moved the GO visualisations to supplementary, Fig.S2.

7. It should be made clear which set of experiments was performed as biological replicates and which did not.

We have added details on the number of replicates used in each experiment. [lines158 and 330]

8. Based on the heatmap in Fig1A, expression of Rax is significant in GFP- cells at days 4 and 5. The authors should comment or discuss this.

We have amended the text and supplemental methods section to include more details of our FACS protocol. The limitations of our sorting procedure include the fact that cells are not sorted into pure GFP expressing and non-expressing populations. Rather the GFP negative sample may contain some cells with low Rax expression or cells that have just begun to express Rax that were not excluded by our sorting. Our aim was to collect sufficient numbers of cells for each condition and separate out cells that expressed GFP to get a more uniform population of cells to study. [line 188]

It is also of note that the heatmap shows Rax expression by day 3. Although it was not detectable by imaging there were around 100 cells per organoid that FACS marked as GFP positive but were

© 2023. Published by The Company of Biologists under the terms of the Creative Commons Attribution License (https://creativecommons.org/licenses/by/4.0/).

retained within the day 3 sample to ensure we had a complete picture of the gene expression at this time point.

9. Ln 99 of materials and methods mentions that the sorting of GFP+ was performed "when possible". The authors should state the differences in the conditions in the different experiments.

This has been expanded to detail exactly how cells were sorted. [line89-100]

10. The sentence closing the first section of the results (Ln 270) is an overstatement and should be moderated. I cannot see how the results shown in this section on their own could reflect and drive solid conclusions on brain cell fate specification.

We agree with the referee and have changed this sentence to:

"In summary, these first analyses of RNA-seq data generated from the timecourse of optic vesicle organoid development, show that this is a robust and relevant model system with which to study the gene dynamics underlying mammalian eye field specification." [line 312]

11. Appropriate citations should be added to back up the argument that opens the second part of the results section (starting Ln 279).

We have added several citations that discuss and review the current knowledge regarding gene regulation via TF-binding at accessible cis-regulatory elements. [line 319-325]

12. Ln 342-343. I suggest being consistent and using the EF-up or EF-down nomenclature on the whole manuscript unless referring to a different subset of genes.

We have modified the text to consistently use "EF-up" or "EF-down" terminology.

13. Ln 692 Refers to Fig.S4F, but this figure has only panels A-D.

This was a typo and has been corrected in the text.

14. Figures 6B and E and the figure legend do not indicate the differences between the panels, or the time stage of the experiments.

The figure legend for Fig6 has been updated to include these details.

CROSS-CONSULTATION COMMENTS

I agree with the comments and suggestions made by the other two reviewers, which identify similar and also specific issues in the manuscript. I believe they are all pertinent and should be acknowledged before re-submitting.

Reviewer #2 (Significance (Required)):

The manuscript by Owen et al, presents the analysis of in vitro eye vesicle organoids derived from mouse ESCs at stages equivalent to when the eye field is specified in vivo. This work is pertinent and necessary as detailed data on gene expression in early eye organoids was missing in the field and is necessary for the interpretation of experiments in these systems.

Although the computational data provided in this manuscript is based on consensus TF motifs, the functional relevance of the specific motifs must be proven before being able to drive any significant conclusions, and one should be moderate about the conclusion that can be driven from this kind of analysis. Still, the analysis put forward is a good reference and starting point for future functional studies. One possible limitation of this study is that the quantification of the expression of genes is based on the RNAseq data, and the expression data should be further confirmed using a proper quantitative method like qPCR.

© 2023. Published by The Company of Biologists under the terms of the Creative Commons Attribution License (https://creativecommons.org/licenses/by/4.0/).

This study will be of interest to the audience studying eye development and disease in animal model systems and humans.

My lab studies the genetic, cellular, and molecular aspects of eye specification, development and disease in zebrafish, and study mutations identified patients with eye globe defects.

Reviewer #3 (Evidence, reproducibility and clarity (Required)):

Studies in Xenopus embryos have established that the specification of the eye field requires a core set of transcription factors (TFs) that impose eye identity to anterior neural plate progenitors. In this manuscript the authors have used mouse embryonic stem cells-derived optic vesicle organoid to ask if the acquisition of mammalian eye identity requires the same set of TFs. They further use different genomic approaches to identify the cis-regulatory elements involved in the expression of these genes and analyses the consequences of altering the sequence of some of the identified regulatory elements. Their results confirm that in mammals the acquisition of eye field identity requires the upregulation of the expression of the same core set of TFs described in Xenopus, with a particularly important role for three of them: Rax, Pax6 and Lhx2. This upregulation is associated to the downregulation of pluripotency genes.

This is a generally well-performed study, that indeed involves a large amount work and adds the identification of several cis-regulatory elements controlling the expression of this core set of already identified eye field TFs. However, conceptually the study does not add much to what is already known and the authors do not offer any very original conclusion from their study. They have generated a large amount of information that likely could allow them to go beyond what is known. For example, they could enlarge the composition of the gene regulatory network that controls eye field specification, given than one of their argument is that their analysis can predict the composition of such a network. Perhaps, they could also address some of the questions that are posed in the discussion. This will strengthen the manuscript and valorize their work.

Additional points that could be taken into consideration are the following:

1) According to the text, the authors identify only 53 CREs with decreased chromatin accessibility (ATACseq signal) between the 3 day and 5 days timepoints, versus the 7752 CREs with increased signal. However, this contrasts with the proportion of genes upregulated/ downregulated in their RNAseq analysis (37 vs 448) and with the notion that specification of the eye field involves the concomitant repression of other neural fates. This also suggests that at least an important fraction of the dynamic ATACseq peaks associated with 161 of the 448 downregulated genes increase their accessibility and allow the recruitment of transcriptional repressors. However, the role of TF binding and chromatin accessibility dynamics on gene repression is poorly discussed and the authors need to provide some interpretation of these observations. Also, authors interpret the fact that the presence of BS for EF downregulated genes, such as En2 and GATA6, correlates with increased chromatin accessibility as a consequence of the fact that TFBS can be bound by different TF paralogs but do not seem to consider that these TFs have been reported to work as transcriptional repressors, so that their downregulation could well explain the changes in chromatin accessibility.

We thank the reviewer for their interesting comments here. We have added short discussions on both main points above (EF-down genes linked to peaks with increasing accessibility and En2/Gata as transcriptional repressors) in the text related to the analysis of our ATAC-seq data. The notion that a loss of repression leading to the activation of gene-expression is indeed a very exciting one and one that we have thought about in the context of the switch-on of the eye-field TFs. This certainly deserves further future work, however in the present study we wanted to be careful not to overinterpret our data. To robustly gain insights into the loss of repression, experiments such as En1/Gata6 ChIP-seq would be very useful, though we are unable to perform these in the near future. [lines428-436]

2) ATACseq signal analysis is an indirect measure of TF binding. The authors demonstrate the predictive nature of this analysis of TF dynamics and have use an available Sox2 ChIP dataset. However, this does not allow assessing dynamic changes in the occupancy of this TF and its correlation with ATACseq. Therefore, at least for few of the TF stressed in this work (e.g. Sox2 and Otx2 and for which good antibodies exist) they could attempt ChIP-seq analysis. This would

© 2023. Published by The Company of Biologists under the terms of the Creative Commons Attribution License (https://creativecommons.org/licenses/by/4.0/).

considerably strengthen the work and provide support to an idea that the authors have particularly emphasized in their manuscript.

We agree with the referee that not having generated ChIP-seq data does not allow us to validate some of the hypotheses and evidence provided by the computational analysis of our ATAC-seq data - we have added a discussion of this limitation in the discussion section of our manuscript [line1163-1171]. We do note however, as observed in Bentsen et al, 2020, that compared to simple TF-motif occurrence analyses, TF-footprinting analyses (such as those we have performed) yield results on putative TF binding that are much closer to more direct measurements of TF binding via e.g. ChIP-seq.

We fully agree that it would be very interesting to perform ChIP-seq/Cut&Cut experiments on the organoid system for a set of interesting TFs identified in our study. Unfortunately, because the lab of Prof FitzPatrick has now closed, it is not possible for us to perform further wet-lab experiments in the very near future.

In an attempt to address this further, we did perform a search of the literature to try to find additional publicly-available ChIP-seq data to support our hypotheses. Unfortunately, we did not find any datasets relevant to the very early stages of eye development (which certainly points to future directions of work). We nevertheless still wanted to see whether there was any evidence that the binding of TFs of interest at the proximal Rax element, was plausible. To do this we explored the UniBind database (Puig et al, 2021), which is a database of robust TF-DNA interactions (specific TF binding sites), which uses a combination of 1000s of publicly-available ChIP-seq datasets alongside machine-learning optimised motif scanning to identify robust TFBS with ChIP evidence. In Fig. S10C, we show, for the Rax proximal element, the UniBind TFBSs for Sox2, Pou5f1, Otx2, Tcf7 and Lhx2 (these were all the relevant TFs to our work), across all experiments/ model-systems accounted for by UniBind. This additional data is highly suggestive that the binding sites we examine within this genomic locus, particularly those for Sox2, Pou5f1 and Otx2 which we are interested in, are indeed true binding sites for the corresponding TFs. Relevant to our discussion on differential occupancy of binding, there are overlapping UniBind TFBS for Pou5f1 and Otx2, beside Sox2 TFBS (also consistent with the main message of the paper by Danno et al., 2008). Although these have been identified in different model systems, it is not unreasonable that they may also serve as binding sites in the organoid model we work with (which at early time points in particular should not be very different from E14 mESC cells). We have updated the text in this section of the manuscript to include our above findings. [line982-1003]

With the exception of performing new ChIP-seq experiments, which we are unable to do, we feel that this is the best we can reasonably extract from the literature, without also over-interpreting our results.

3) Previous studies (i.e. 10.1242/dev.067660; 10.1093/hmg/ddt562) have shown the importance of gene dosage in eye field specification and repression of other fates. These studies could be included in the discussion, which, in its current version is a quite brief and leaves out many of the reported analysis.

We thank the referee for pointing us to this very relevant question - we have added this to the further research questions in the discussion. [line1184-1189]

CROSS-CONSULATION COMMENTS

The comments from the other reviewers complement the aspects that we have underscored and should be fully considered as they will contribute to improve the manuscript.

Reviewer #3 (Significance (Required)):

This is a generally well-performed study, that indeed involves a large amount work and adds the identification of several cis-regulatory elements controlling the expression of this core set of already identified eye field TFs. However, conceptually the study does not add much to what is already known and the authors do not offer any very original conclusion from their study. They

© 2023. Published by The Company of Biologists under the terms of the Creative Commons Attribution License (https://creativecommons.org/licenses/by/4.0/).

have generated a large amount of information that likely could allow them to go beyond what is known.

Developmental neurobiologists, genome

Analyses that we are not able to carry out

The reviewers have pointed out that some additional laboratory work could be performed to further check and support our findings and the conclusions we present in our manuscript. While we are confident that the experimental work included in this paper is robust (and furthermore we have both been very careful not to overstate our findings as well as provide further evidence and data in our response to address concerns raised by the reviewers), we are unfortunately not in a position to do any further laboratory work. The reason for this is that Prof. FitzPatrick has retired from his position at the MRC HGU and his research group has closed. Where possible, we have made a significant effort to add further evidence to hypotheses we suggest using publicly-available data (as discussed above in the point-by-point response).

Second decision letter

MS ID#: DEVELOP/2022/201432

MS TITLE: Characterization of an Eye Field-like State during Optic Vesicle Organoid Development

AUTHORS: Liusaidh J Owen, Jacqueline Rainger, Hemant Bengani, Fiona Kilanowski, David R FitzPatrick, and Andrew Sofronis Papanastasiou

Apologies for the length of time it took to obtain reviews on your revised manuscript. One of the original referees did not manage to submit a review and so I have made a decision based on the two reviews I have received. You will be pleased to see that both referees are happy for the manuscript to be published in Development and there is only one minor comment for you to consider. Additionally, your manuscript is longer than our normal word length and so please consider reducing word length if possible. The referees' comments are appended below, or you can access them online: please go to BenchPress and click on the 'Manuscripts with Decisions' queue in the Author Area.

Reviewer 1

Advance summary and potential significance to field

The authors have address part of the concerns that have been raised in the first revision. The manuscript provides useful and important information that deserve publication.

Comments for the author

The authors have address part of the concerns that have been raised in the first revision. The manuscript provides useful and important information that deserve publication.

Reviewer 2

Advance summary and potential significance to field

The manuscript by Owen et al, presents the analysis of in vitro eye organoids derived from mouse ESCs at stages equivalent to when the eye field is specified in vivo. This work is pertinent and necessary, as detailed data on gene expression in early eye organoids was missing in the field and is required for the interpretation of experiments in these systems.

© 2023. Published by The Company of Biologists under the terms of the Creative Commons Attribution License (https://creativecommons.org/licenses/by/4.0/).

The computational data provided in this manuscript is based on consensus TF motifs and de novo discovery, and the analysis put forward is a good reference and starting point for future functional studies. This study will be of interest to the audience studying eye development and disease in animal model systems and humans.

Comments for the author

The authors have taken on board most of my comments and suggestions, and I think the manuscript is much improved compared to the original version. The additions and improvements have dissipated the concerns in my first revision. I would just suggest using the current nomenclature tcf7l1 instead of tcf3, which is also used for mice as well as in humans in current databases like Ensembl.

Second revision

Author response to reviewers' comments

We sincerely thank the referees for re-reading our manuscript and for their positive comments to our manuscript changes.

Regarding the change of name from Tcf3 to Tcf7l1, we didn't feel this was the correct thing to do. Our labelling of genes comes from the Ensembl database and in the mouse genome these are two separate genes (both shown to have roles in the developing neuroectoderm). Instead, given the importance of the Tcf-family of genes in regulating beta-catenin/Wnt-signalling and the general similarity of binding motifs of this group of genes, we thought it to be most scientifically correct to analyse all the related transcription factors (Tcf3, Tcf7, Tcf7l1, Tcfl2) in our manuscript. We have updated the text to reflect this and feel this provides a more unbiased and complete presentation for these genes. We note that Tcf7l1 does not have a mouse TF-PWM in the JASPAR database.

We thank all referees once again for their critical and thoughtful comments on our manuscript -- these have helped to significantly improve the presentation and scientific content of our article.

Yours sincerely,
Dr. Andrew Papanastasiou

Third decision letter

MS ID#: DEVELOP/2022/201432

MS TITLE: Characterization of an Eye Field-like State during Optic Vesicle Organoid Development

AUTHORS: Liusaidh J Owen, Jacqueline Rainger, Hemant Bengani, Fiona Kilanowski, David R FitzPatrick, and Andrew Sofronis Papanastasiou
ARTICLE TYPE: Techniques and Resources Article

Thank you for sending your manuscript to Development through Review Commons

I am happy to tell you that your manuscript has been accepted for publication in Development, pending our standard ethics checks.

© 2023. Published by The Company of Biologists under the terms of the Creative Commons Attribution License (https://creativecommons.org/licenses/by/4.0/).
